# REST promotes ETS1‐dependent vascular growth in medulloblastoma

**DOI:** 10.1002/1878-0261.12903

**Published:** 2021-02-07

**Authors:** Shavali Shaik, Shinji Maegawa, Amanda R Haltom, Feng Wang, Xue Xiao, Tara Dobson, Ajay Sharma, Yanwen Yang, Jyothishmathi Swaminathan, Vikas Kundra, Xiao Nan Li, Keri Schadler, Arif Harmanci, Lin Xu, Vidya Gopalakrishnan

**Affiliations:** ^1^ Department of Pediatrics University of Texas, MD Anderson Cancer Center Houston TX USA; ^2^ Department of Pediatrics University of Texas Southwestern Medical Center Dallas TX USA; ^3^ Department of Population & Data Sciences University of Texas Southwestern Medical Center Dallas TX USA; ^4^ Quantitative Biomedical Research Center University of Texas Southwestern Medical Center Dallas TX USA; ^5^ Departments of Abdominal Imaging and Cancer Systems University of Texas MD Anderson Cancer Center Houston TX USA; ^6^ Department of Pediatrics Northwestern University Feinberg School of Medicine Chicago IL USA; ^7^ Center for Precision Health School of Biomedical Informatics The University of Texas Health Science Center Houston TX USA; ^8^ Department of Molecular and Cellular Oncology University of Texas, MD Anderson Cancer Center Houston TX USA; ^9^ Center for Cancer Epigenetics University of Texas, MD Anderson Cancer Center Houston TX USA; ^10^ Brain Tumor Center University of Texas, MD Anderson Cancer Center Houston TX USA

**Keywords:** medulloblastoma, REST, NRSF, tumor microenvironment, vasculature

## Abstract

Expression of the *RE1*‐silencing transcription factor (REST), a master regulator of neurogenesis, is elevated in medulloblastoma (MB) tumors. A cell‐intrinsic function for REST in MB tumorigenesis is known. However, a role for REST in the regulation of MB tumor microenvironment has not been investigated. Here, we implicate REST in remodeling of the MB vasculature and describe underlying mechanisms. Using *REST^TG^* mice, we demonstrate that elevated *REST* expression in cerebellar granule cell progenitors, the cells of origin of sonic hedgehog (SHH) MBs, increased vascular growth. This was recapitulated in MB xenograft models and validated by transcriptomic analyses of human MB samples. REST upregulation was associated with enhanced secretion of proangiogenic factors. Surprisingly, a REST‐dependent increase in the expression of the proangiogenic transcription factor E26 oncogene homolog 1, and its target gene encoding the vascular endothelial growth factor receptor‐1, was observed in MB cells, which coincided with their localization at the tumor vasculature. These observations were confirmed by RNA‐Seq and microarray analyses of MB cells and SHH‐MB tumors. Thus, our data suggest that REST elevation promotes vascular growth by autocrine and paracrine mechanisms.

## Graphical abstract

Expression of REST, a negative regulator of neurogenesis, is aberrantly elevated in SHH medulloblastomas and contributes to tumor progression through upregulation of hedgehog signaling and cell proliferative pathways. Here, we identify a role for REST in vascular remodeling. REST elevation increased secretion of proangiogenic factors and upregulated genes with potential to drive an endothelial cell‐like phenotype in tumor cells.

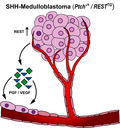

**Abbreviations**
ANG: angiogenin
ANGPT1: angiopoietin‐1
ANGPT2: angiopoietin‐2
BLI: bioluminescence imaging
CCL2 / MCP1: C‐C motif chemokine ligand 2 / monocyte chemoattractant protein‐1
CGNPs: cerebellar granule neuron progenitors
CXCL16: C‐X‐C motif chemokine ligand 16
CXCL8 / IL‐8: C‐X‐C motif chemokine ligand 8 / interleukin‐8
ETS1: E26 oncogene homolog 1
H&E: hematoxylin and eosin
HBMEC: human brain microvascular endothelial cell
HUVEC: human umbilical vein endothelial cell
IHC: immunohistochemistry
MB: medulloblastoma
NS/P: neural stem/progenitor
PBS‐T: phosphate buffered saline/Tween‐20
PGF: placental growth factor
PLAU / uPA: plasminogen activator, urokinase / urokinase‐type plasminogen activator
PLG: angiostatin/plasminogen
REST / NRSF: RE1‐silencing transcription factor / neuron‐restrictive silencer factor
SHH: sonic hedgehog
THBS1 / TSP1: thrombospondin‐1
THBS2 / TSP2: thrombospondin‐2
TME: tumor microenvironment
VEGFR1: vascular endothelial growth factor receptor‐1
VM: vascular mimicry / vasculogenic mimicry
WNT: Wingless

## 1 Introduction

Medulloblastoma (MB) is the most common malignant brain tumor in children and frequently occurs in the cerebellum [1, 2, 3]. MBs are classified into Wingless (WNT), sonic hedgehog (SHH), Group 3, and 4 molecular subgroups [4, 5]. Although patients with WNT‐driven MBs have good prognosis, subsets of patients with SHH tumors and most with Group 3/4 tumors have poor outcomes [1, 6]. The underlying reasons are not well understood. Cerebellar granule neuron progenitors (CGNPs) are thought to be the cells of origin of SHH‐MB tumors [7]. The SHH signaling pathway is frequently deregulated in SHH‐driven MBs, and its activation promotes CGNP hyperproliferation [8]. This added to the lack of their terminal neuronal differentiation, which contributes to MB development [9, 10].

Aberrations in chromatin remodeling are believed to drive MB tumors [11, 12]. Our previous work showed elevated expression of the RE1‐silencing transcription factor (REST), a transcriptional repressor of neuronal differentiation genes, in human MB tumors, and found it to be correlated with poor patient prognosis [6, 10, 13, 14]. REST's contribution to MB genesis was demonstrated through the generation of a novel transgenic mouse model (*REST^TG^*), where human REST transgene could be conditionally expressed in CGNPs [10]. Compared with age‐matched wild‐type (WT) mice, *REST^TG^* animals exhibited an expanded external granule layer (EGL), where CGNPs reside [10]. *Ex vivo*‐cultured CGNPs from *REST^TG^* mice also showed poorly neurogenesis, suggesting that REST increases cell proliferation and blocks differentiation [10]. In the background of constitutive activation of SHH signaling (*Ptch^+/−^)*, *REST^TG^* mice developed poorly differentiated tumors with 100% penetrance, accelerated kinetics of 10–90 days, and leptomeningeal dissemination when compared to *Ptch^+/−^* mice, which highlighted a cell‐intrinsic role for the protein in tumor progression [10].

The tumor microenvironment (TME) plays an important role in tumorigenesis. Angiogenesis and vasculogenesis, which are important for the growth, progression, and metastasis of tumors, are controlled by an imbalance between pro‐ and antiangiogenic molecules that are secreted by endothelial cells, tumor cells, or other cells present in the TME [15, 16, 17, 18, 19, 20]. These vessels are frequently structurally and functionally abnormal [17]. Brain tumor vasculature growth can occur through mechanisms such as co‐option, angiogenesis, vasculogenesis, vascular mimicry (VM), and tumor endothelial differentiation [21, 22]. Abnormal vasculature in MBs has also been noted. For example, clusters of abnormal, thick‐walled arterial‐type vessels along with numerous variably joined small venous and capillary structures are seen in WNT‐MBs [23]. In SHH‐MBs, increased expression of proangiogenic factors has been described [24]. Functional studies have attributed a role for SHH ligand‐dependent stimulation of tumor stromal secretion of placental growth factor (PGF) and neuropilin (NRP) in SHH‐MB development [18].

Here, we describe a role for REST in the control of MB vasculature. Employing a combination of transgenic and xenograft mouse models, analyses of publicly available transcriptomic data on human MB tumors, and functional studies, we demonstrate that REST drives increased expression of proangiogenic molecules, vascular endothelial growth factor (VEGF), and PGF. *In vivo*, tumors in *Ptch^+/−^/REST^TG^* mice and animals bearing human MB xenografts exhibit a significant increase in the number and size of blood vessels compared with control mice. Interestingly, REST elevation is also associated with increased expression of vascular endothelial growth factor receptor‐1 (VEGFR1) and the proangiogenic transcription factor, E26 oncogene homolog 1 (ETS1), in CGNPs of *REST^TG^* mice compared with cells from WT cerebella. Human MB tumors engineered to express REST transgene had increased expression of molecules identified as ‘VEGF pathway genes’ by RNA‐Seq analyses and colocalized with endothelial cells *in vitro* and *in vivo*, suggesting that REST elevation promotes angiogenesis‐related gene expression changes in MB cells. Our studies are the first to implicate REST, a canonical regulator of neurogenesis, in the control of MB vasculature.

## 2 Materials and methods

### 2.1 Cell culture

Human MB cells (DAOY, DAOY‐REST (DAOY‐R), UW426, UW426‐REST (UW426‐R), UW228, UW228‐REST (UW228‐R), and D283) and mouse v‐Myc transformed neural stem cell (C17.2) and its isogenic derivative expressing human (h) REST transgene (ST2) were cultured as described previously [6, 10]. CGNPs were isolated from WT and *REST^TG^* mice and cultured as previously outlined [10]. Human umbilical vein endothelial cells (HUVEC; Cat# CC‐2519; Lonza, Alpharetta, GA, USA) were cultured in endothelial growth medium‐2 (EGM‐2) with recommended growth factors (Cat# CC‐3162; Lonza). Human brain microvascular endothelial cells (HBMEC; Cat# HEC02) and endo growth medium (EGM; Cat# MED001) were purchased from Neuromics (Edina, MN, USA) and cultured in complete medium contained EGM‐2 and EGM (4 : 1 ratio). 293T cells were grown in Dulbecco's Modified Eagle's Medium in the absence of serum.

### 2.2 *In vivo* assays

Animal experiments and procedures were done following approval by the Institutional Animal Care and Use Committee. DAOY or DAOY‐R cells (50 ,000 cells in 3 μL) stably expressing firefly luciferase (ffluc) were implanted into cerebella of 4‐ to 6‐month‐old NOD/SCID gamma null (NSG; NOD.Cg‐Prkdcscid Il2rgtm1Wjl/SzJ) mice (The Jackson Laboratory, Bar Harbor, ME, USA), using a guide screw [25]. Tumor growth was monitored by bioluminescence imaging (BLI) using the Caliper Life Sciences IVIS Spectrum IVIS200 *in vivo* imaging system (Caliper LIfe Sciences, Hopkinton, MA, USA) [10]. Mice were euthanized when signs of morbidity were noted [10]. Brains were collected and sectioned for IHC analysis. *REST^TG^* mice and *Ptch^+/−^/REST^TG^* were generated as described [10].

### 2.3 Immunohistochemistry

Brains from WT, *REST^TG^, Ptch^+/−^, Ptch^+/−^/REST^TG^* mice, and animals bearing DAOY/DAOY‐R xenografts and high‐REST and low‐REST (LR/HR) patient‐derived orthotopic xenografts (PDOX), obtained from X. Nan Li (under a material transfer agreement), were formalin‐fixed and embedded in paraffin. 4‐μm‐thick brain sections were cut and used for IHC analysis. Deparaffinized, rehydrated sections were subjected to heat‐mediated antigen retrieval in citrate buffer / Tris‐EDTA buffer, quenched, and incubated with primary antibodies to CD31 (Cat# DIA310; Dianova GmbH, Hamburg, Germany; Cat# ab28364; Abcam, Cambridge, MA, USA), VEGFR1 Cat# AMAB90703; Sigma‐Aldrich, St. Louis, MO, USA), and ETS1 (Cat# ab26096; Abcam) at 4 °C, overnight. After washing, sections were incubated with secondary antibody conjugated to horseradish peroxidase (HRP) (Cat# 115‐035‐003 and 115‐035‐006; Jackson ImmunoResearch, West Grove, PA, USA) and developed using 3,3′‐diaminobenzidine substrate (Cat# SK‐4100; Vector Laboratories, Burlingame, CA, USA). Hematoxylin and eosin (H&E) counterstaining was then done. Stained sections were visualized under a microscope (Nikon ECLIPSE E200; Melville, NY, USA) and images acquired using an Olympus SC100 camera (Waltham, MA, USA). Images were processed using CellSens Entry imaging software (Olympus Life Sciences, Waltham, MA, USA). CD31‐positive blood vessels were quantified, and statistical analyses were performed among different groups.

### 2.4 Immunofluorescence

Paraffin‐embedded brain sections were deparaffinized and rehydrated in a Gemini AS auto‐stainer (Thermo Fisher Scientific, Waltham, MA, USA), and washed in PBS, and antigen retrieval was performed using eBioscience™ IHC Antigen Retrieval Solution—High pH (Cat# 00‐4956‐58; Thermo Fisher Scientific, Pittsburgh, PA, USA) by heating to 98 °C for 15 min in LabVision™ PT module™ (Thermo Fisher Scientific, Pittsburgh, PA, USA). Slides were placed in distilled water, washed with 0.1% Tween‐20 in PBS (PBS‐T), blocked in 1% FBS in PBS for 1 h, and then coincubated with goat anti‐luciferase (1 : 150, Cat# NB100‐1677SS; Novus Biologicals, Centennial, CO, USA) and mouse anti‐CD31 (1 : 250, Cat# Dia‐310; DIANOVA GmbH). After washing in PBS‐T, slides were incubated with donkey anti‐goat (1 : 300, Cat# 705‐586‐147) and donkey anti‐rat (1 : 300, Cat# 712‐546‐153; Jackson ImmunoResearch) antibodies for 1 h. Washed slides were mounted in Fluorogel II mounting media and visualized under a fluorescence microscope.

### 2.5 *In vitro* angiogenesis assay

*In vitro* angiogenesis assay (tube assay) was performed by first placing matrigel (Cat# 354230; Thermo Fisher Scientific, Waltham; 100 μL/well) in 96‐well sterile culture plates [26]. HUVEC (5 × 10^4^ cells/25 μL) cells were mixed with endothelial medium and with conditioned medium derived from DAOY/ DAOY‐R/, UW228/ UW228‐R, UW426/UW426‐R (1 : 1 ratio), placed on the matrigel, and incubated in a CO_2_ incubator at 37 °C. In other experiments, HBMECs were incubated with conditioned medium from DAOY‐R cells transduced with lentivirus expressing shRNA against *ETS1* (shETS1‐1) or a nonspecific sequence (shControl). After 16 h, cells were incubated with Calcein‐AM (Cat# C3100MP; Thermo Fisher Scientific, Waltham) for 30 min and rinsed with the endothelial cell culture medium. The number of tubes formed in matrigel was determined by fluorescence microscopy and image analysis/quantification as described [26]. To distinguish between colocalized MB and endothelial cells, MB cells and HBMECs cells were loaded with cell tracker red and green (Cat#s C34552 and C2925; Invitrogen, Carlsbad, CA, USA), respectively. Cells were coincubated in matrigel for 16 h followed by fluorescence microscopy and quantification of tube formation and colocalization.

### 2.6 qRT‐PCR

RNA was extracted from MB cell lines using Quick‐RNA MiniPrep Kit (Cat# D4008; Zymo Research, Irvine, CA, USA). Equal amounts of RNA were reverse‐transcribed into cDNA using the iScript cDNA Synthesis Kit (Bio‐Rad, Hercules, CA, USA), and qRT‐PCR was performed in triplicate as described [10]. Relative mRNA expression, normalized to 18*S* ribosomal RNA, was determined by the comparative $2^{‐\Delta \Delta C_{t}}$ method. Normalized mRNA expression was graphed as fold change compared with parental cell line.

Primer sequences are as follows:

*hREST*‐Forward: *5’‐GGCAGCTGCTGTGATTACCT‐3’*

*hREST*‐Reverse: *5’‐AGTTGTTATCCCCAACCGGC‐3’*

*h18s*‐Forward: *5’‐CGGCGACGACCCATTCGAAC‐3’*

*h18s*‐Reverse: *5’GAATCGAACCCTGATTCCCCGTC‐3’*

### 2.7 Western blot analysis

Cell lysates from human and mouse MB cells and CGNPs from WT and *REST^TG^* mice brains were prepared in EBC lysis buffer [26]. Samples were subjected to SDS/PAGE and western blot analyses with the following primary antibodies: REST (Cat# 07579; Millipore, Billerica, MA, USA), VEGFR1 (Cat# 2479), ETS1 and alpha‐tubulin (Cat#s 14069 and 9099, respectively; Cell Signaling Technology, Danvers, MA, USA), and beta‐actin (Cat# ab40742; Abcam). After washing and incubation with the corresponding HRP‐conjugated secondary antibodies (Jackson ImmunoResearch), membranes were developed using SuperSignal (Cat# 34075 and Cat# 34087; Thermo‐Scientific, Waltham, MA) followed by autoradiography.

### 2.8 Lentivirus production and transduction

Lentiviral constructs expressing GFP and shRNAs against *ETS1* were purchased from institutional shRNA and ORFeome Core. Lentiviral particles were prepared by cotransfection of 293T cells with plasmids pax2 and MD2 using OptiMEM®I and Lipofectamine® 2000 Reagent (Cat#s 31985‐062 and 11668‐019, Thermo Fisher Scientific, Waltham). DAOY‐R cells were transduced with shRNA control or shETS1‐expressing lentivirus for 72 h, and GFP‐positive cells were sorted by flow cytometry. *ETS1* knockdown was confirmed by western blotting, and cells were used in *in vitro* angiogenesis assays. Nucleotide sequences for the shRNAs are as follows:

*shETS1‐1: 5′‐CTTGATATGGTTTCACATC‐3′*

*shETS1‐2: 5′‐TAATTGATACCCGGCCCTG‐3′*

*shControl: 5′‐ATCTCGCTTGGGCGAGAGT‐3′*

### 2.9 Human angiogenesis proteome profiler assay

Proteome profiler human angiogenesis array (Cat# ARY007; R&D Systems Minneapolis, MN, USA) was used to measure levels of pro‐ and antiangiogenic molecules using conditioned media from DAOY, UW228, UW426/UW426‐R, and D283 cells. Quantification was done by imagej analysis (https://imageJ.nih.gov).

### 2.10 RNA‐Seq analyses

The quality of sequencing reads was evaluated using NGS QC Toolkit (v2.3.3) [27], and high‐quality reads were extracted. The human reference genome sequence and gene annotation data, hg38, were downloaded from Illumina iGenomes. (https://support.illumina.com/sequencing/sequencing_software/igenome.html). The quality of RNA‐sequencing libraries was estimated by [28] mapping the reads onto human transcript and ribosomal RNA sequences (Ensembl release 89) using Bowtie (v2.3.2). STAR (v2.5.2b) [29] was employed to align the reads onto the human and viral genomes. SAMtools (v1.9) [30] was employed to sort the alignments, and HTSeq Python package was employed to count reads per gene [31]. DESeq2 R Bioconductor package was used to normalize read counts and identify differentially expressed (DE) genes [31, 32]. KEGG [33] pathway data were downloaded using KEGG API (https://www.kegg.jp/kegg/rest/keggapi.html), and gene ontology (GO) data were downloaded from NCBI FTP (ftp://ftp.ncbi.nlm.nih.gov/gene/DATA/gene2go.gz). The enrichment of DE genes to pathways and GOs was calculated by Fisher's exact test in R statistical package.

### 2.11 Gene expression profile in MB patient samples and MB cell lines

Microarray data sets containing gene expression values of MB tumors were obtained from Gene Expression Omnibus (www.ncbi.nlm.nih.gov/geo). The GSE85217 data set, which contains Affymetrix Human Gene 1.1 ST Array profiling of 763 primary MB samples, was used to evaluate gene expression. Microarray data were normalized using the robust multiarray average method. The expression data for each gene were *Z*‐score‐transformed. Hierarchical clustering based on the expression of neuronal differentiation markers divided the 223 SHH‐type MB patient samples into six distinct clusters (Clusters 1–6) as described previously [10]. We also analyzed the publically available data sets (GSE86574, GSE107405, GSE37382, GSE50765) in GEO and from the data set provided by Cho *et al*., in R2: Genomics Analysis and Visualization Platform (hgserver1.amc.nl/cgi‐bin/r2/main.cgi) for gene expression analysis [34, 35, 36, 37].

### 2.12 Statistical analysis

The experimental data reported are mean ± SD of a minimum of three samples. *P*‐value of < 0.05 was considered to be statistically significant. *P*‐values for comparisons between every pairwise combination among clusters (1–6) based on gene expression status were obtained using the unpaired *t*‐test with Welch's correction using the graphpad prism version 7.0 (GraphPad, San Diego, CA, USA). Significance is indicated as *P < 0.05, **P < 0.01, ***P < 0.001, or ****P < 0.0001; where necessary for clarity, lack of significance is indicated (ns). Student's *t*‐test and ANOVA were performed for significance between groups.

## 3 Results

### 3.1 *REST^TG^* mice exhibit increased cerebellar vasculature

**Fig. 1:**
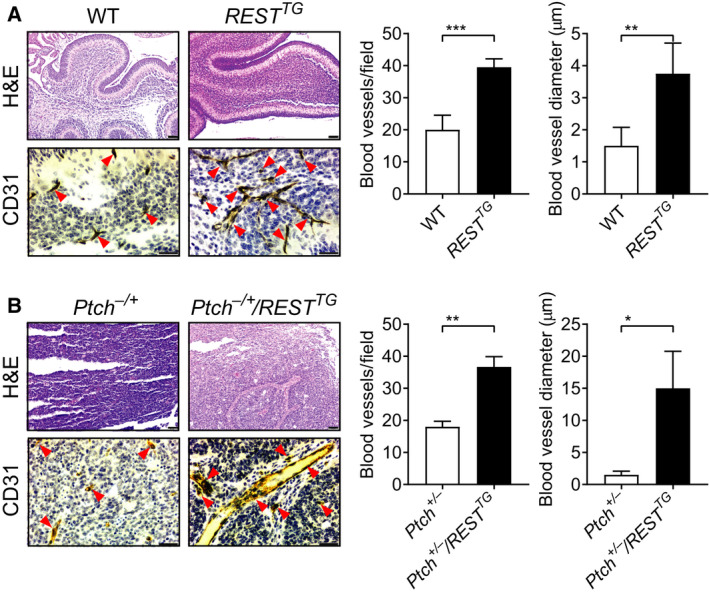
REST promotes vasculature in *REST^TG^* cerebella and *Ptch^+/−^/REST^TG^* tumors. H&E staining and IHC for CD31 were performed on (A) cerebellar sections from WT and *REST^TG^* mice and (B) tumor sections from *Ptch^+/−^* and *Ptch^+/−^/REST^TG^* transgenic mice, to demonstrate the vasculature changes (left panels). Arrowheads show the blood vessels. Quantitation of blood vessels in sections (*n* = 3; three fields /section), and average blood vessel diameter is shown in the right panels for A & B. Scale bars in A, B; H&E = 20 μm; CD31 = 10 μm. Data show individual variability and means ± SD. *P*‐values were obtained using Student's *t*‐test. *P < 0.05, **P < 0.01, ***P < 0.001.

We had previously shown that conditional REST elevation in CGNPs caused an abnormal expansion of the cerebellar EGL in *REST^TG^* mice compared with WT animals [10]. A more careful examination of H&E‐stained sections revealed an increased presence of vascular structures in the cerebella of *REST^TG^* mice compared with WT mice (Figs 1A and S1A). These observations were confirmed by IHC, which revealed a twofold increase in CD31‐positive vessels in the cerebella of *REST^TG^* mice relative to that in WT animals (Fig. 1A). A significant increase in lumen diameter and branching was also seen in the cerebella of *REST^TG^* mice compared with WT cerebella (Figs 1A and S1A). These findings suggest a REST‐dependent increase in cerebellar vasculature.

### 3.2 REST elevation drives tumor vasculature

A role for REST in the progression of SHH‐driven MBs was first described by our previous work, where we showed that in *Ptch^+/−^* mice with constitutive activation of SHH signaling, REST elevation (*Ptch^+/−^/REST^TG^*) promoted tumors with 100% penetrance, accelerated kinetics of 10–90 days, and leptomeningeal dissemination [10]. To determine whether REST elevation also contributed to modulation of tumor vasculature, IHC assessment of CD31 staining of tumor‐bearing cerebella of *Ptch^+/−^* and *Ptch^+/−^/REST^TG^* mice was performed. While H&E staining showed larger and more infiltrative tumors in *Ptch^+/−^/REST^TG^* mice in contrast to *Ptch^+/−^* mice, CD31 staining confirmed a twofold increase in blood vessels in *Ptch^+/−^/REST^TG^* tumors compared with *Ptch^+/−^* tumors (Figs 1B and S1B). Once again, *Ptch^+/−^/REST^TG^* tumors exhibited a demonstrable increase in the number of vessels and vessel diameter relative to tumors in *Ptch^+/−^* mice (Figs 1B and S1B).

**Fig. 2:**
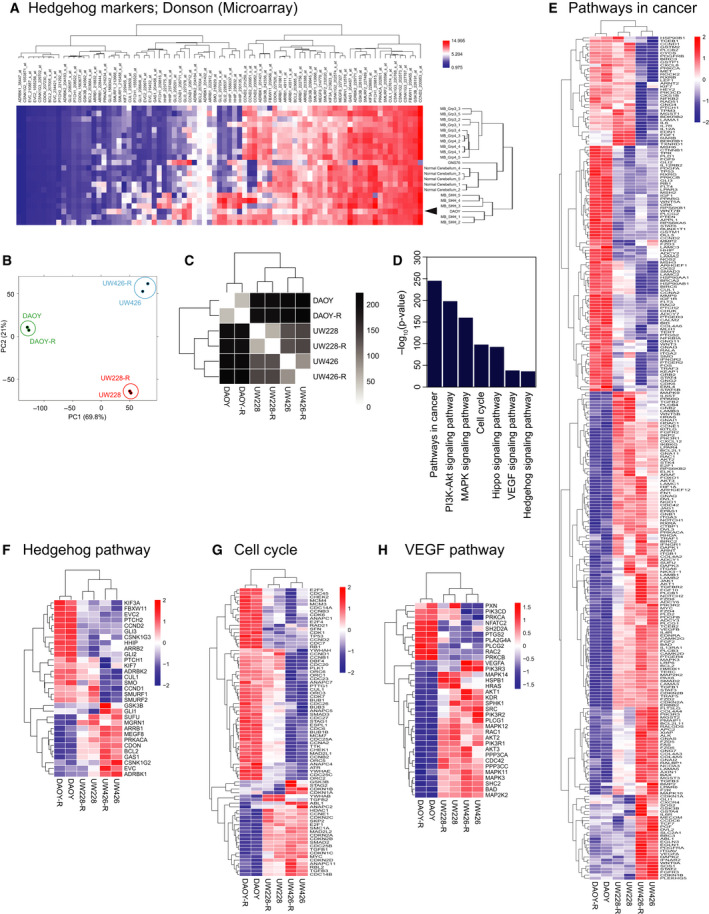
Gene expression profiling of human SHH‐MB cell lines demonstrates expression of hedgehog pathway markers. (A) Unsupervised hierarchical cluster analysis of gene expression involving hedgehog pathway in GSE86574. We isolated the expression profiles of normal cerebellum (*n* = 5), MB_SHH (*n* = 5), MB_Grp3 (*n* = 5), MB_Grp4 (*n* = 5), and two MB cell lines (DAOY and ONS76) from GSE86574, and performed hierarchical clustering assay. Expression values were *Z*‐score‐transformed. Red, high expression; blue, low expression. Arrowhead shows the position of DAOY clustered with MB_SHH patient samples. (B) Principal component analysis of gene expression landscape from LR/HR isogenic pairs of the three MB cell lines (DAOY/DAOY‐R, UW228/UW228‐R, and UW426/UW426‐R). (C) Hierarchical clustering analysis of gene expression landscape from DAOY/DAOY‐R, UW228/UW228‐R, and UW426/UW426‐R cells. (D) Enriched KEGG pathways on differentially expression genes (FDR adjusted *P* < 0.05) between LR/HR isogenic pairs of MB cell lines. The plot shows negative log 10‐transformed *P*‐values for the enriched pathways. (E‐H) Heatmap of DE genes in pathways in cancer (E), hedgehog signaling (F), cell cycle regulation (G), and VEGF signaling (H).

We also validated our findings from genetically engineered mice in human MB cells. As a first step, we performed RNA‐Seq analyses of three commonly used MB cell lines, DAOY, UW228, and UW426, to compare their gene expression profile with that of published transcriptomic data (GSE86574) from normal cerebellum, human SHH, Group 3, and Group 4 MB tumors, as well as ONS76 MB cell line. Using data from GSE86574, we performed hierarchical clustering analysis of expression of genes involved in hedgehog pathway. DAOY cells clustered with SHH‐MBs, indicating significant similarity in their expression profiles with respect to hedgehog pathway markers with that of SHH‐MBs (Fig. 2A). In addition, we also observed clustering of DAOY cells with SHH‐MBs using subtype‐specific marker genes used for NanoString analyses [38, 39] (Fig. S2A,B). These 22 subtype‐specific genes and 30 hedgehog marker genes were sufficient to divide a cohort of 763 MB patient tumors into the four MB subgroups (Fig. S2C,D) [4]. In addition, a recent study showed the expression profile of DAOY cells to be similar to that of SHH‐MB patient tumors by hierarchical clustering assay using a 22 genes NanoString panel [40]. These data provide direct transcriptomic proof that DAOY cells are representative of SHH‐MBs. We also analyzed the expression profiles of the above 22 subtype‐specific genes in another published data set (GSE107405) and in our RNA‐Seq data, to show that DAOY, UW228 and UW426 cells clustered separately from cell lines derived from Group 3 or Group 4 MB patients (Fig. S2E,F) [35]. With respect to most of 22 subtype‐specific marker genes, these MB cell lines showed‐ expression patterns that were similar to SHH‐MB subtype patient tumors (Fig. S2G–J). Collectively, these results confirm that DAOY, UW228, and UW426 cells retain features of SHH‐MBs.

To study the effect of constitutive REST expression on the biology of DAOY, UW228, and UW426 cells, and specifically with respect to genes involved in vascular development, we generated LR/HR isogenic pairs of the three MB cell lines and performed RNA‐sequencing analysis. Interestingly, we observed that all three isogenic pairs of MB cell lines retain their overall gene expression landscape even after overexpressing REST (Fig. 2B,C), suggesting that altered REST expression influences a restricted number of biologically relevant genes rather than creating global expression changes. To further functionally characterize these genes whose expression is modulated by REST elevation, we conducted pathway enrichment analysis and defined KEGG pathways that are enriched for REST‐driven gene expression changes (Fig. 2D). These enriched genes defined pathways with roles in cancer development (Fig. 2E), hedgehog signaling (Fig. 2F), cell cycle regulation (Fig. 2G), VEGF signaling (Fig. 2H), hippo signaling (Fig. S3A), and MAPK signaling (Fig. S3B).

**Fig. 3:**
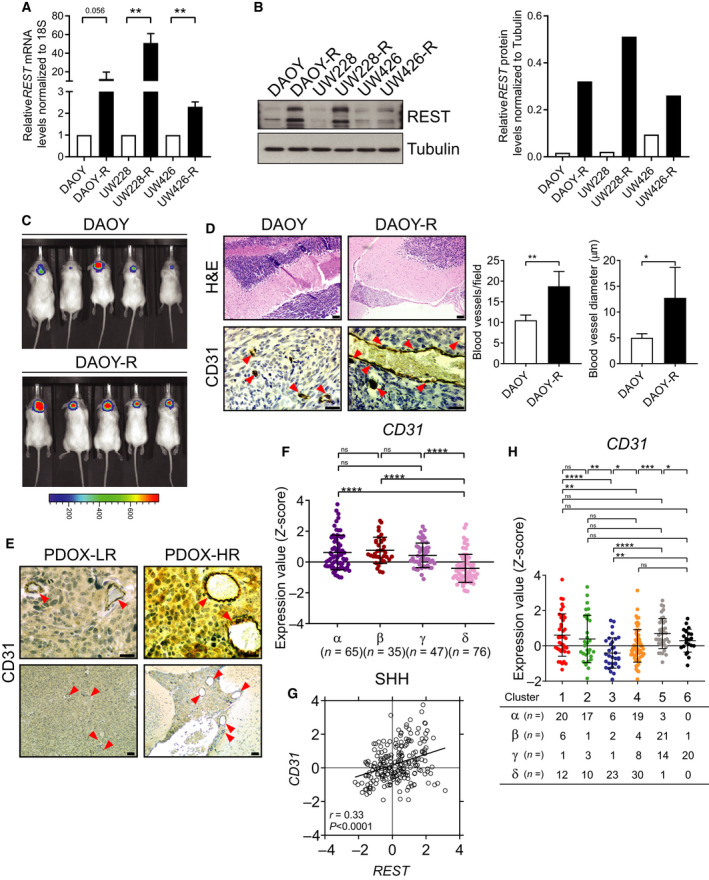
Human MBs with REST elevation have increased vasculature. (A, B) Relative REST *mRNA* and protein levels in DAOY/DAOY‐R, UW228/UW228‐R, and UW426/UW426‐R cells were measured by RT‐PCR and western blot analysis, respectively. Right panel in B shows quantification data. Tubulin served as a loading control. Data show individual variability and means ± SD. *P*‐values were obtained using Student's *t*‐test. *P < 0.05, **P < 0.01, ***P < 0.001, ****P < 0.0001. (C) Growth of DAOY‐ffluc and DAOY‐R‐ffluc cells implanted in the cerebella of immunodeficient mice (*n* = 5) was monitored by BLI. Color scale bar indicates luminescence intensity across the images. (D) Brain sections from the above mice were studied by H&E (top panel) to locate tumors, and IHC for CD31 (bottom panel) to identify tumor vasculature. Arrowheads show the blood vessels. Quantitation of blood vessels and average blood vessel diameter is shown in the right panel (*n* = 3; three fields/section). Scale bars: H&E = 20 μm; CD31 = 10 μm. (E) IHC for CD31 in tumor‐bearing brain sections of PDOX mice (*n* = 3., three fields/section) to demonstrate vasculature changes in tumors (left panels). Arrowheads show the blood vessels. Scale bars: top (40×) = 10 μm, bottom (10×) = 20 μm. (F) *CD31* mRNA expression profile in four subtypes of SHH‐MB patient samples measured by microarray (α; *n* = 65, β; *n* = 35, γ; *n* = 47, δ; *n* = 76) from GSE85217 data set [4]. Each dot corresponds to one individual patient. Data show individual variability and means ± SD. *P*‐values were obtained using the unpaired *t*‐test with Welch's correction. ns, not significant. *P < 0.05, **P < 0.01, ***P < 0.001, ****P < 0.0001. (G) Scatter plot of correlation of *REST* mRNA expression and *CD31* mRNA expression. Figure shows the plot across all 223 SHH‐MB patients (*r* = 0.33, *P* < 0.0001). (H) *CD31* mRNA expression profile in SHH‐MB patient samples. Hierarchical clustering based on expression of neuronal differentiation markers divided the SHH‐MB patient samples into six distinct clusters (Cluster 1; *n* = 39, Cluster 2; *n* = 31, Cluster 3; *n* = 32, Cluster 4; *n* = 61, Cluster 5; *n* = 39, Cluster 6; *n* = 21) [10]. Each dot corresponds to an individual patient. Data show individual variability and means ± SD. ns, not significant. *P*‐values were obtained using the unpaired *t*‐test with Welch's correction. *P < 0.05, **P < 0.01, ***P < 0.001, ****P < 0.0001.

Based on our data described in Figs 1 and 2, we further investigated the possibility that REST elevation contributes to modulation of vasculature. For this, DAOY and DAOY‐REST (DAOY‐R) cell lines engineered to express firefly luciferase (ffluc) were injected into the cerebellum of NOD/SCID mice (*n* = 5, each) and tumor growth was monitored by BLI. REST expression in these cells was confirmed by RT‐PCR and Western blotting (Fig. 3A,B). Although both DAOY and DAOY‐R cells formed tumors, the latter grew more rapidly and formed larger tumor masses at the time of euthanasia (72 days) (Figs 3C and [Link], [Link]). Brains were harvested from both cohort of animals, sectioned, and studied by H&E staining, and IHC using anti‐CD31 antibody, to identify tumors and vasculature, respectively (Figs 3D and [Link], [Link]A). Quantitation of CD31‐positive structures showed a twofold increase in the number and diameter of vessels in DAOY‐R tumors compared with DAOY tumors (Fig. 3D, right panel). Similar differences in vasculature were also noted in sections of mice brains bearing HR‐ and LR‐PDOX tumors (Figs 3E and [Link], [Link]B).

*CD31* expression was also quantified in a publicly available transcriptome database (GSE85217) of human SHH‐MB samples [4]. As seen in Fig. 3F, *CD31* gene expression was higher in SHH‐α, β, γ tumor samples compared with SHH‐δ tumors. *CD31* and *REST* expression showed a strong overall positive correlation (*r* = 0.33, *P* < 0.0001) in SHH subgroup of MB samples and in SHH‐β (*r* = 0.36, *P* = 0.04), γ (*r* = 0.40, *P* = 0.005) subtypes (Figs 3G and Fig S10A). SHH‐MBs were also divided into six clusters, based on the expression of REST target neuronal differentiation genes, where a significant increase in *REST* mRNA levels was seen in clusters 1, 2 (SHH‐α), and 5 (SHH‐β) tumors [10]. The pattern of *CD31* expression paralleled that of *REST*, with higher levels seen in clusters 1, 2, and 4, relative to clusters 3, 4, and 6 (Fig. 3H). These findings were confirmed using GSE37382 and GSE50765 data sets [37]. Finally, comparisons of tumor samples with normal cerebella made using GSE data sets revealed upregulation of CD31 in MB samples and a positive correlation with REST expression in SHH‐MBs (Fig. S6A–C). Thus, the above observations indicate that REST supports tumor vasculature in mice and its expression in subsets of SHH tumors is strongly correlated with the expression of CD31 (Fig. S10A–D).

### 3.3 REST elevation promotes secretion of proangiogenic factors in MBs

**Fig. 4:**
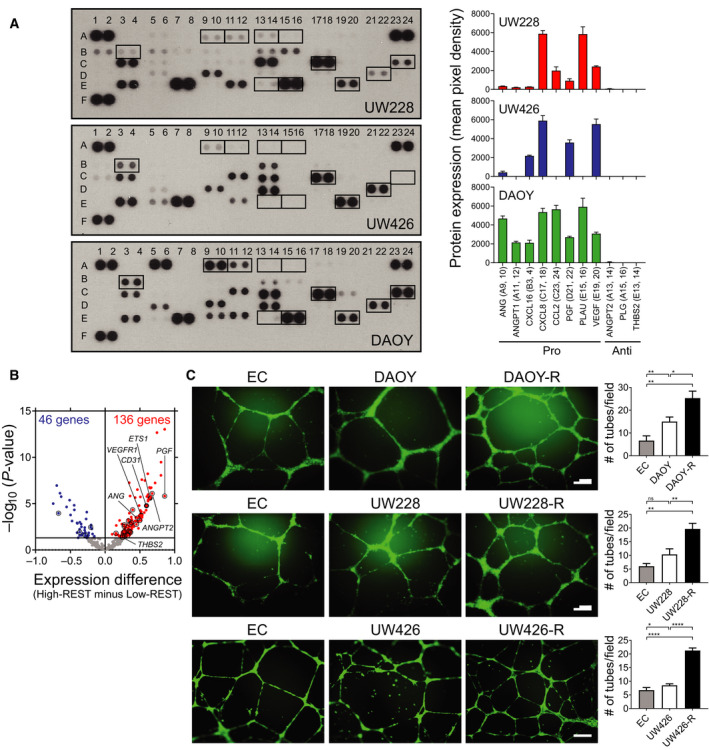
REST elevation promotes secretion of proangiogenic factors. (A) Conditioned culture medium from UW228, UW426, and DAOY cells was used to probe a commercially available human angiogenesis array for a panel of known angiogenesis‐related secreted proteins (left panel). The boxes and box numbers represent pro‐ or antiangiogenic proteins and correspond to molecules listed in Table S1. Densitometric quantitation is shown on the right (also see Table S1). (B) Volcano plots show angiogenesis‐related genes DE between SHH‐MB samples (GSE85217) with high‐ and low‐REST expression [4]. Samples with HR expression (*n* = 117); α; *n* = 42/65 (64.6%), β; *n* = 28/35 (80.0%), γ; *n* = 15/47 (31.9%), δ; *n* = 32/76 (42.1%). Samples with low‐REST expression (*n* = 106); α; *n* = 23/65 (35.4%), β; *n* = 7/35 (20.0%), γ; *n* = 32/47 (68.1%), δ; *n* = 44/76 (57.9%). The gene expression difference between HR/LR samples is shown on the *x*‐axis, the *P*‐value (in –log_10_ scale) on the *y*‐axis. The horizontal line indicates a *P*‐value of 0.05. Circles represent genes encoding proteins analyzed in A (also see Tables S2 and S3). Black dots identify expression levels for *CD31*, *VEGFR1,* and *ETS1*. (C) *In vitro* angiogenesis assay to show relative tube formation by HUVECs cultivated in endothelial culture (EC) alone or with conditioned media from DAOY/DAOY‐REST(R), UW228/UW228‐REST(R), and UW426/UW426‐REST(R) cell cultures. EC medium and conditioned medium were used in a 1 : 1 ratio. Tubes formed in matrigel were photographed after 16 h (left panel) and quantitated (right panel). Data shown are mean ± SD. *P*‐values were obtained using Student's *t*‐test. *P < 0.05, **P < 0.01, ****P < 0.0001*, n* = 3. Scale bar; 100 μm.

To investigate whether REST expression modulated secreted pro‐ or antiangiogenic factors, we incubated a Proteome Profiler Human Angiogenesis Array membrane with antibodies that can detect the presence of up to 55‐ human angiogenesis‐related secreted proteins‐ in conditioned media from DAOY, UW228, UW426, and D283 cells. Densitometry was used to quantitate levels of these secreted proteins (Figs 4A and [Link], [Link]A; Table S1). DAOY and UW228 cells expressed proangiogenic molecules, angiogenin (ANG), angiopoietin‐1 (ANGPT1), C‐X‐C motif chemokine ligand 16 (CXCL16), IL‐8 (CXCL8), MCP1 (CCL2), PLGF (PGF), uPA (PLAU), and VEGF. The levels of ANG, ANGPT1, CXCL16, CCL2, and PGF were higher in DAOY than in UW228 cells and paralleled REST levels in these cells (Fig. 4A) [10]. The above proangiogenic molecules, except ANGPT1, CCL2, and PLAU, were also expressed in UW426 cells (Fig. 4A). ANGPT1, CXCL8, PGF, and PLAU were also detected in D283 cells, a Group 3/4 MB cell line (Fig. S7A). Antiangiogenic molecules angiopoietin‐2 (ANGPT2), angiostatin‐2 (PLG), and thrombospondin‐2 (TSP2/THBS2) were not detected in DAOY, UW228, and UW426 cells (Fig. 4A). REST dependency for the above changes was established by comparing the secretome of UW426 and UW426‐REST cells, which confirmed an increase in the levels of the proangiogenic molecules and ligands for VEGFR1 VEGF and PGF under conditions of REST elevation (Figs 4A and [Link], [Link]B).

Gene expression microarray data derived from SHH‐MBs also confirmed these findings [4]. SHH‐MB samples were divided into two groups (117 HR and 106 LR tumors) based on the average *Z*‐score of their REST expression. The expression of 410 angiogenesis‐related genes listed in Table S2 was studied between these two cohorts using a volcano plot, and genes with significantly differential expression were identified (*P* < 0.05) (Fig. 4B). Of these, 136 genes showed higher expression and 46 genes showed lower expression in samples with higher REST levels (Table S3). Of the 11 proteins examined in Fig. 4A, genes encoding *ANG*, *ANGPT2*, *PGF, THBS2, CD31, VEGFR1, and ETS1* had significantly higher expression in HR MBs (Fig. 4B).

Finally, *in vitro* angiogenesis/tube formation assay was carried out using conditioned media from isogenic pairs of DAOY/DAOY‐R, UW228/UW228‐R, and UW426/UW426‐R cells. HUVECs labeled with cell tracker green dye were placed on matrigel and incubated with the conditioned media from the above isogenic pairs of cells to monitor tube formation. Fluorescence microscopy showed that conditioned medium from DAOY and DAOY‐R cells supported a twofold and threefold increase in tube formation, respectively, relative to control HUVECs cultivated in unconditioned growth medium (Fig. 4C). Similar REST‐dependent increases in tube formation were noted with UW228/UW228‐R and UW426/UW426‐R cells (Fig. 4C). Together, these data support a role for REST elevation in promoting angiogenesis *in vitro*.

### 3.4 REST elevation drives ETS1‐dependent increase in VEGFR1 expression

**Fig. 5:**
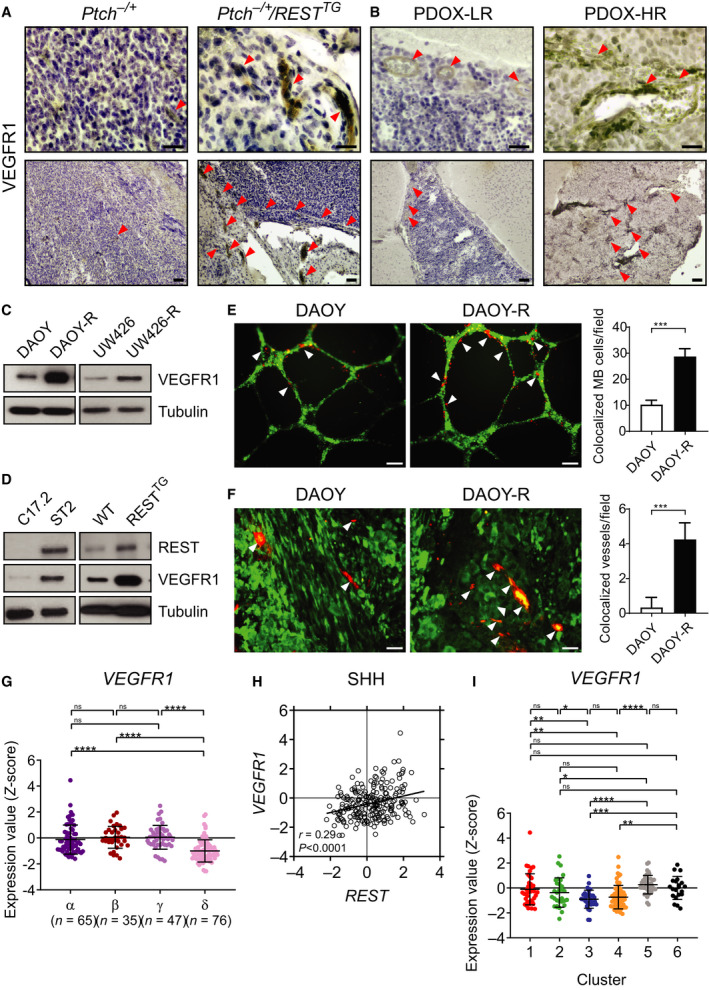
MBs with elevated *REST* expression upregulate *VEGFR1* and colocalize with endothelial cells. (A‐B) IHC was performed on cerebellar sections (*n* = 3) from *Ptch^+/−^* and *Ptch^+/−^/REST^TG^* mice and PDOX animals (*n* = 3) to demonstrate VEGFR1 expression. Arrowheads show blood vessels; Scale bars: top (40×) = 10 μm, bottom (10×) = 20 μm. (C‐D) Western blot analysis to measure VEGFR1 levels in human DAOY/DAOY‐R and UW426/UW426‐R cells, and VEGFR1 and REST protein levels in mouse C17.2/ST2 cells, and in CGNPs from WT/*REST^TG^* mice. Tubulin served as a loading control. (E) HUVECs were cocultured with DAOY or DAOY‐R cells on matrigel, and tube formation was assessed after 16 h. DAOY/DAOY‐R are in red, while HUVECs are shown in green color. Quantitation of the relative numbers of DAOY or DAOY‐R cells colocalized with HUVECs is shown on the right. (F) Immunofluorescence assay to show colocalization in yellow of CD31‐positive endothelial cells (red) and luciferase‐positive tumor cells (green) in tumor sections from DAOY or DAOY‐R xenografts. Quantitative data (*n* = 3, three fields/section) is shown on the right. *P*‐values were obtained using Student's *t*‐test. ***P < 0.001. (G) Profile of *VEGFR1* mRNA expression in microarray data of four subtypes of human SHH‐MB samples from GSE85217 data set [4]. Each dot corresponds to one individual patient. Data show individual variability and means ± SD. *P*‐values were obtained using the unpaired *t*‐test with Welch's correction. ns, not significant. *P < 0.05, **P < 0.01, ***P < 0.001, ****P < 0.0001. (H) Scatter plot of correlation of *REST* mRNA expression and *VEGFR1* mRNA expression. Figure shows the plot across all 223 SHH‐MB patients (*r* = 0.29, *P* < 0.0001). (I) *VEGFR1* mRNA expression profile in SHH‐MB patient samples. Hierarchical clustering based on expression of neuronal differentiation markers divided the SHH‐MB patient samples into six distinct clusters [10]. Each dot represents an individual patient. Data show individual variability and means ± SD. *P*‐values were obtained using the unpaired *t*‐test with Welch's correction. ns, not significant. *P < 0.05, **P < 0.01, ***P < 0.001, ****P < 0.0001.

We next asked whether the levels of VEGFR1, a cognate receptor for PGF and VEGF, are modulated in a REST‐dependent manner. IHC showed that tumors in *Ptch^+/−^*/*REST^TG^* mice and animals with HR‐PDOX had higher VEGFR1 expression compared with *Ptch^+/−^* and LR‐PDOX, respectively (Figs 5A,B and S8A,B). Western blotting of cell lysates from DAOY/DAOY‐R and UW426/UW426‐R cell pairs showed a clear enhancement of VEGFR1 levels in the higher REST context compared with the parental cells (Fig. 5C). VEGFR1 levels were also similarly increased in ST2 cells with constitutive *hREST* expression, relative to parental C17.2 cells (Fig. 5D, left panel). Likewise, relative to WT CGNPs, cells from *REST^TG^* mice exhibited higher VEGFR1 protein levels (Fig. 5D, right panel). Tubulin served as a loading control for these assays (Fig. 5C,D).

Then, a role for REST in tube formation was confirmed by co‐incubating cell tracker green‐labeled HBMCs with cell tracker red‐labeled DAOY/DAOY‐R cells. Surprisingly, REST elevation was associated with a significant increase (threefold) in the co‐localization of MB cells and endothelial cells (Fig. 5E). Indeed, co‐immunofluorescence staining of ffluc‐expressing DAOY and DAOY‐R tumor sections from NSG mice, with anti‐CD31 and anti‐luciferase antibodies, also revealed a fourfold increase in REST‐dependent colocalization (yellow) of tumor cells (green) with endothelial cells (red) (Fig. 5F). These findings raise the possibility that REST elevation in MB cells could lead to an endothelial cell‐like phenotype or VM, a phenomenon in which cancer cells form blood vessels independent of, or in association with endothelial cells in tumors [41, 42].

Like *CD31, VEGFR1* gene expression was significantly higher in SHH‐α, SHH–β, SHH–γ MBs compared with SHH‐δ tumors, and *VEGFR1* and *REST* expression showed a strong overall positive correlation (*r* = 0.29, *P* < 0.0001) in SHH subgroup of MB samples and in SHH‐γ (*r* = 0.30, *P* = 0.04) and SHH‐δ subtypes (*r* = 0.23, *P* = 0.047) (Figs 5G,H and S10E). A trend toward significance was noted in SHH‐α (*r* = 0.22, *P* = 0.07) and SHH‐β (*r* = 0.31, *P* = 0.07) (Fig. S10E). Higher *VEGFR1* expression was observed in clusters 1, 2, 5, and 6 compared with clusters 3 and 4 in the differentiation‐based grouping of tumor samples (Fig. 5I). Among WNT, Group 3, and Group 4 MB tumor samples, a positive correlation between *REST* and *VEGFR1* was detected when all Group 4 tumors were collectively considered (*r* = 0.16, *P* = 0.005), but a statistically significant correlation could not be detected in the individual subtypes of Group 4 tumors (Figs 5G,H and S10E–H). These results indicate that VM may be unique to SHH‐MBs, although the limited availability of subgroup/subtype information in other patient tumor data sets precluded further investigation of this observation (Fig. S8C,D).

**Fig. 6:**
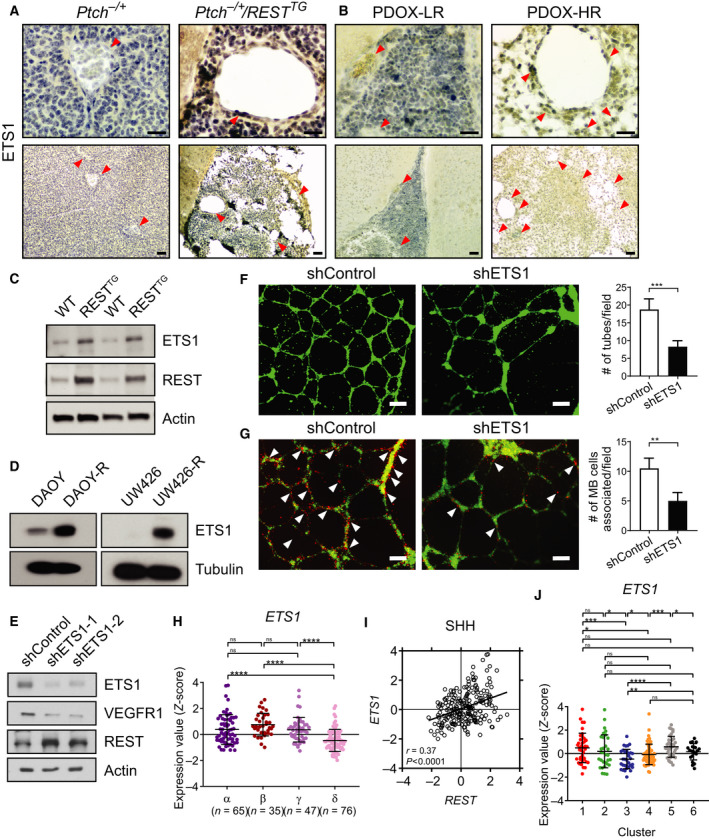
REST upregulates *ETS1* expression. IHC was performed on cerebellar sections from (A) *Ptch^+/−^* and *Ptch^+/−^/REST^TG^* transgenic mice (*n* = 3) and (B) PDOX (*n* = 3) to demonstrate ETS1 expression. Arrowheads show blood vessels. Scale bars: top (40×) = 10 μm, bottom (10×) = 20 μm. Western blot analysis to measure ETS1 and REST protein levels in (C) CGNPs from WT/*REST^TG^* mice, (D) ETS1 levels in human DAOY/DAOY‐R and UW426/UW426‐R cells, and (E) ETS1, VEGFR1 and REST levels in shControl or shETS1‐1/shETS1‐2 expressing DAOY‐R cells. Tubulin and actin were used as loading controls. (F) *In vitro* tube formation assay to assess tube formation by HBMECs was done by culturing with conditioned medium from shControl or shETS1‐1 expressing DAOY‐R cells for 16 h in matrigel (left panels). Quantitation of tube formation is shown on the right. Three fields were counted per group. *P*‐values were obtained using Student's *t*‐test. ***P < 0.001, Scale bar; 100 μm. (G) *In vitro* tube formation assay to assess tube formation by HBMECs was done by coculturing HBMEC and shControl or shETS1‐1 expressing DAOY‐R cells for 16 h in matrigel (left panels). Quantitation of colocalization of DAOY‐R cells with HBMECs following ETS1 knockdown (using anti‐shETS1‐1—left panels) is shown on the right. *P*‐values were obtained using Student's *t*‐test. **P < 0.01. Scale bar; 100 μm. (H) Profile of *ETS1* mRNA expression in microarray data of four subtypes of human SHH‐MB samples from GSE85217 data set [4]. Each dot represents a patient. Data show individual variability and means ± SD. *P*‐values were obtained using the unpaired *t*‐test with Welch's correction. ns, not significant. *P < 0.05, **P < 0.01, ***P < 0.001, ****P < 0.0001. (I) Scatter plot of correlation of *REST* mRNA expression and *ETS1* mRNA expression. The figure shows the plot across all 223 SHH‐MB patients (*r* = 0.37, *P* < 0.0001). (J) *ETS1* mRNA expression profile in SHH‐MB patient samples. Hierarchical clustering based on the expression of neuronal differentiation markers divided the SHH‐MB patient samples into six distinct clusters [10]. Each dot represents a patient. Data show individual variability and means ± SD. *P*‐values were obtained using the unpaired *t*‐test with Welch's correction. ns, not significant. *P < 0.05, **P < 0.01, ***P < 0.001, ****P < 0.0001.

If MBs with REST elevation do indeed mimic endothelial cells, we reasoned that transcription factors directing endothelial specification such as ETS1 may also be expressed in tumor cells [43]. Again, IHC confirmed that *Ptch^+/−^*/*REST^TG^* mice and HR‐PDOX brain sections had higher ETS1 expression relative to *Ptch^+/−^,* and LR‐PDOX samples, respectively (Figs 6A,B and S9A,B). Western blotting performed with lysates from WT/*REST^TG^* CGNPs, as well as DAOY/DAOY‐R and UW426/UW426‐R cells, showed an increase in ETS1 levels, which paralleled REST levels, showing that *ETS*1 expression was REST‐dependent (Fig. 6C,D). The above findings taken in conjunction with *VEGFR1* being a known ETS1 target gene, led us to assess whether REST‐dependent increase in *VEGFR1* expression in DAOY‐R cells is mediated by ETS1 [44, 45]. Knockdown experiments showed that reduction of *ETS1* in DAOY‐R cells using two different shRNA‐*ETS1* constructs did indeed result in a decrease in VEGFR1 levels in these cells (Fig. 6E). REST levels remained unaffected, relative to actin controls. *In vitro* angiogenesis assays showed that a reduction in *ETS1* expression in DAOY‐R caused a significant decline in tube formation, as well as a decrease in tumor endothelial cell colocalization at the tubes, confirming the involvement of ETS1 in this process under conditions of REST elevation (Fig. 6F,G).

**Fig. 7:**
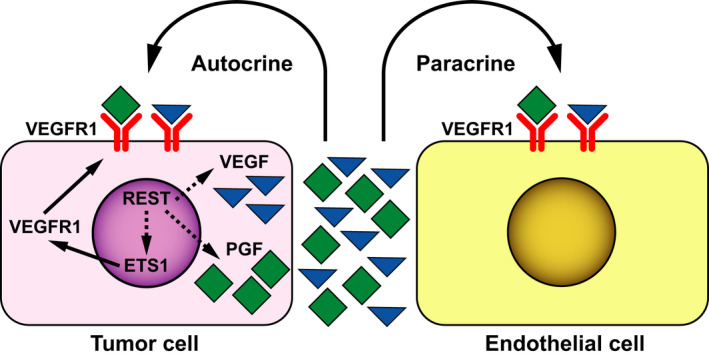
Model to show autocrine and paracrine effects of REST on MB vasculature. REST expression in MB cells promotes increased secretion of proangiogenic molecules such as VEGF and PGF, ligands that activate the VEGFR1 receptor present on vascular endothelial cells. In addition, REST upregulates the expression of ETS1 and VEGFR1 in MB cells to mimic endothelial cell behavior. Dotted lines indicate that the mechanism(s) are unclear. Both processes contribute to an increase in vasculature and tumor progression.

*ETS1* expression was also studied using bulk transcriptomic data from human SHH‐MB samples described in Figs 2, 3, 4. First, *ETS1* expression was significantly higher in SHH‐α, SHH–β, SHH–γ tumor samples compared with SHH‐δ tumors (Fig. 6H). *ETS1* and *REST* expression showed a strong overall positive correlation (*r* = 0.37, *P* < 0.0001) in SHH subgroup of MB samples and in SHH‐β (*r* = 0.40, *P* = 0.02), SHH–γ (*r* = 0.43, *P* = 0.003), and SHH–δ (*r* = 0.32, *P* = 0.005) subtypes (Figs 6I and S10I). When SHH‐MBs were divided into the six neurogenesis‐based clusters, a significant increase in *ETS1* mRNA levels was seen in clusters 1, 2, 5, and 6, relative to clusters 3 and 4 (Fig. 6J). Among WNT, Group 3, and Group 4 MB tumor samples, a positive correlation between *REST* and *ETS1* was detected in Group 4 tumors (*r* = 0.31, *P* < 0.0001), with a significant correlation seen in Group 4 α (*r* = 0.38, *P* < 0.0001), β (*r* = 0.27, *P* = 0.005) and γ (*r* = 0.35, *P* < 0.0001) tumor subtypes (Fig. S10J‐L). Collectively, the above data suggest that REST‐dependent modulation of tumor vasculature is ETS1‐dependent, with a positive association between *REST* and *ETS1* expression seen in subsets of SHH and Group 4 tumors (Fig. S10I‐L). We also detected positive correlations between *REST* and *ETS1* expression in GSE50765 and GSE37382 [37] sample sets, respectively, but not in the data set provided by Cho *et al*. [36], likely due to the limitations and/or differences of probe sets and sample numbers among patient data sets (Fig. S9C,D). Overall, our data suggest that REST contributes to MB vasculature through cell‐extrinsic and cell‐intrinsic mechanisms (Fig. 7).

## 4 Discussion

REST is a canonical regulator of neurogenesis and plays a key role during normal brain development. It is this aspect of REST function that has been most widely studied and reported in the literature [14, 46]. REST binds to the *RE1* sequence found in the regulatory regions of many neuronal genes to silence their expression. REST controls neural development by regulating neural lineage specification. It promotes neural stem/progenitor (NS/P) self‐renewal while restricting their maturation into neurons [47]. REST shows differential expression during neural development, with its levels being highest in embryonic stem cells (ES) and gradually declining thereafter as cells transition through NS/P cells into mature neurons [47]. However, its expression is maintained in cells destined for glial specification, suggesting that REST levels may dictate other neural lineage choices [46, 48]. Although most studies have focused on its function in neural cells, genome‐wide chromatin occupancy studies have identified a reasonable number of potential REST target genes, which are not involved in neural development [49]. Abnormal REST activity is implicated in the genesis of many neural cancers including MB, glioblastoma, diffuse intrinsic pontine glioma (DIPG) and neuroblastoma [6, 10, 26, 50, 51]. Its aberrant expression in these cancers has been associated with poor patient survival [6, 26, 51]. However, these studies were also mostly focused on the‐cell‐intrinsic functions of REST and attributed roles for the protein in the control of cell proliferation and/or blockade of neural lineage specification during tumor development [10].

The TME, which encompasses the vascular network, stromal cells, immune cells, extracellular matrix, and fibroblasts, plays a key role in tumor growth and progression [52]. Cell–cell communication between tumor cells and TME also influence tumor response to therapies [53]. For these reasons, the crosstalk between cancer cells and the TME has been a subject of intense research in many cancers. However, similar studies in pediatric brain cancers have been quite limited and mostly restricted to the study of vascular networks and their role in tumor progression and metastasis [17, 18, 54]. As stated above, although computational studies have suggested that the REST network may include genes implicated in modulation of the TME, very few follow‐up functional studies have been conducted. REST has been shown to control pericyte biology in‐ Ewing's sarcoma, a primitive neuro‐ectodermal tumor that occurs mostly in adolescents [55, 56]. Our group was ‐ the first to demonstrate a role for the REST‐gremlin axis in controlling the vasculature of DIPG tumors [26]. In the current study, we provide the first demonstration ‐ that REST elevation controls MB vasculature. Indeed, RNAseq analyses‐ showed changes in hippo and MAPK signaling and confirmed our previous findings that REST elevation drives cell proliferation and represses PTCH expression in the more immature SHH‐α MBs, and surprisingly in the more differentiated SHH‐β tumors [10, 57]. We also show that REST controls endothelial cell biology and MB vasculature in part by paracrine mechanisms. VEGF, VEGF165, PDGFA, VEGF121, Ang‐1 (ANGPT1), Ang‐2 (ANGPT2), VEGFC, TGFA, VEGF189, and VEGFB were some of the proangiogenic molecules previously described in MB tumors [24, 58]. Here, we found PGF, ANG, ANGPT1, CXCL8, and CXCL16 to be secreted by MB cell lines. PGF produced by the cerebellar stroma in SHH tumors signals through neuropilin‐1 and promotes MB cell survival [18]. Although levels of antiangiogenic molecules were significantly lower in MB cell lines, this was not recapitulated in human MB samples. Our work is also the first to suggest a role for ETS1 in REST‐dependent angiogenesis in SHH‐MBs. Most importantly, our findings suggest that REST‐driven modulation of tumor vasculature may contribute to the increased incidence of metastasis and poor survival in patients with SHH‐α and SHH‐β MBs. ETS1 is a transcription factor and is a known regulator of angiogenic growth factors such as VEGFR1 [44, 45, 59]. Given the strong correlative parallels in *REST* and *ETS1/CD31/VEGFR1* expression between SHH‐MBs and Group 4 MBs, similar mechanisms could be operational in these two subgroups of MBs. However, this needs further evaluation.

In addition to angiogenesis, brain tumors utilize other mechanisms to acquire new blood vessels, including co‐option, vasculogenesis, and intussusception [17]. Plasticity of cancer cells enables them to mimic endothelial cells, thus leading to the formation of vessels [60]. This has been described in glioblastomas where stem‐like cells were found to differentiate into endothelial cells, and harbored the same genomic alterations as cancer cells [41]. In a study by Wang *et al*. [60] ~ 22% of MB tumors (*n* = 41) were shown to exhibit VM and was also associated with poorer clinical outcomes. Thus, there is support for VM in MBs, although mechanisms have not been defined. Our data suggest that REST elevation in tumor cells may promote VM by driving VEGFR1 expression and possibly activating the protein kinase C alpha pathway [42] (Fig. 7). However, the REST‐VM connection needs to be further investigated.

Antiangiogenic therapies have been under consideration for pediatric and adult brain tumors [61]. For recurrent glioblastoma multiforme (GBM), the median overall survival was 8.63 months for patients treated with bevacizumab, an anti‐VEGF antibody, and 8.91 months when bevacizumab was combined with irinotecan, a chemotherapeutic agent [62] Thus, this study showed bevacizumab alone is beneficial for GBM. Interestingly, recent clinical trials have also demonstrated the efficacy of bevacizumab for the treatment of recurrent MB when combined with chemotherapeutic agents temozolomide and irinotecan or with stereotactic radiosurgery [63, 64]. Despite this promise, clinical use of antiangiogenic agents has not evolved [19, 65]. The development of resistance to anti‐VEGF therapies could be an underlying reason [66]. VM may be yet another cause since angiogenesis inhibitors appear to block the formation of vessels by endothelial cells, but not those originating from tumor cells [67]. Therefore, targeting drivers of VM such as REST or ETS1 may alleviate resistance to conventional angiogenesis inhibitors and need to be further evaluated in preclinical studies. Our previous preclinical studies have demonstrated the feasibility of targeting REST activity through inhibition of associated chromatin remodeling enzymes—G9a/GLP, LSD1, and HDAC1/2 [13, 14, 68]. However, their effect on tumor vasculature was not studied. Downregulation of ETS proteins is correlated with regression of hyaloid vessel endothelial cells [69]. In newborn mice, administration of YK‐4‐279, an inhibitor of ETS and ETS‐related gene activity, decreased the number of hyaloid vessels [69]. YK‐4‐279 was also been shown to reduce tube formation by HUVECs *in vitro*, in a VEGFR1‐dependent manner [69]. Targeting ETS1 for proteolysis, by inhibiting the activity of its deubiquitylase USP9X, may be another interesting strategy, which should be explored [70]. Overall, antiangiogenesis approaches remain under‐investigated for brain tumor therapy.

## 5 Conclusion

The current study is the first to attribute a role for REST in the regulation of MB vasculature. We have provided evidence that its elevation promotes increased secretion of pro‐angiogenic factors, which allows vascular growth. In addition, MB cells with elevated REST expression display molecular and functional features of endothelial cells, suggesting that REST may alter cell fate decisions in MBs by modulating the expression of transcription factors that control angiogenesis, although mechanistic details remain to be delineated (Fig. 7). Targeting REST and ETS1 for the therapeutic modulation of tumor angiogenesis is a topic for future studies.

## Conflict of interest

The authors declare no conflict of interest.

## Author contributions

SS and SM were involved in conceptualization and performance of *in vitro* and *in vivo* experiments, data analysis, and generation of the manuscript. AH, JS, TD, and AS were involved in *in vivo* xenograft experiments, immunofluorescence, and BLI. YY was involved in *in vitro* studies, cell transfection, and microscopy. KS provided guidance on angiogenesis assays. AH, FBW, LX, and XX performed analyses of the RNA‐Seq data. XNL provided reagents. VK provided guidance on imaging analyses and manuscript review. VG was involved in study conceptualization, experimental design, data analysis, funding support, and writing of the manuscript and overall supervision of the project.

## Consent for publication

Consent was obtained from all authors for publication.

## Supporting information


**Fig. S1**. REST promotes vasculature in *REST^TG^* cerebella and *Ptch^+/−^/REST^TG^*. (A) Cerebellar sections from WT and *REST* *^TG^* mice and (B) tumor sections from *Ptch* *^+/‐^* and *Ptch* *^+/‐^* */REST* *^TG^* transgenic mice to demonstrate the vasculature changes. Arrowheads show the blood vessels. (n=3). Scale bars; H&E=20 μm; CD31=10 μm.

**Fig. S2**. Gene expression profiles of subtype specific markers and hedgehog markers in MB cell lines and MB patients. (A) Unsupervised hierarchical cluster analysis of gene expression data using NanoString 22 genes in GSE86574. Expression values were *Z*‐score transformed. Red; high expression, blue; low expression. Arrowhead shows the position of DAOY clustered with MB_SHH patient samples. (B) Unsupervised hierarchical cluster analysis of gene expression data using NanoString 100 genes in GSE86574. (C) Unsupervised hierarchical cluster analysis of gene expression data using 33 hedgehog pathway related genes in publicly available microarray data [4]. (D) Unsupervised hierarchical cluster analysis of gene expression data using NanoString 22 genes [38] in GSE85217 [4]. (E) Unsupervised hierarchical cluster analysis of gene expression data using NanoString 22 genes in GSE107405 [35]. (F) Unsupervised hierarchical cluster analysis of gene expression data using NanoString 22 genes in our RNA‐seq data (Shaik). (G‐J) Gene expression profiles of subtype specific markers (NanoString 22 genes) (WNT, SHH, Group3 and Group4) in GSE85217 [4], GSE107405 [35] and our RNA‐seq data (Shaik). Data show individual variability and means ± SD. *P*‐values were obtained using the unpaired *t*‐test with Welch’s correction. ns, not significant. *P < 0.05, **P < 0.01, ***P < 0.001, ****P < 0.0001.

**Fig. S3**. Heatmaps of differentially expressed genes in hippo signaling pathway and MAPK signaling pathway.

**Fig. S4**. Human MBs with REST elevation have increased vasculature. H&E staining on brain sections from DAOY and DAOY‐REST (DAOY‐R) mice xenografts. *n* = 3. Scale bar; 100 μm. Arrowheads show tumor area.

**Fig. S5**. REST promotes vasculature in DAOY‐R and PDOX‐HR tumors. (A) DAOY/DAOY‐R tumors and (B) PDOX‐LR and PDOX‐HR tumors to demonstrate the vasculature changes. Arrowheads show the blood vessels. (*n* = 3). Scale bars in A; H&E = 20 μm; CD31 = 10 μm. Scale bars in B: top (40×) = 10 μm, bottom (10×) = 20 μm.

**Fig. S6**. REST and CD31 expression in MB patients. (A) *REST* mRNA expression profile in MB patient samples measured by microarray (GSE85217 [4], GSE37382 [37] and Pomeroy’ data sets [36]). Each dot corresponds to one individual patient. Data show individual variability and means ± SD. *P*‐values were obtained using the unpaired *t*‐test with Welch’s correction. ns, not significant. *P < 0.05, **P < 0.01, ***P < 0.001, ****P < 0.0001. (B) *CD31* mRNA expression profile in MB patient samples measured by microarray (GSE85217 [4], GSE37382 [37] and Pomeroy’ data sets [36]). Each dot corresponds to one individual patient. Data show individual variability and means ± SD. *P*‐values were obtained using the unpaired *t*‐test with Welch’s correction. ns, not significant. *P < 0.05, **P < 0.01, ***P < 0.001, ****P < 0.0001. (C) Scatter plot of correlation of *REST* mRNA expression and *CD31* mRNA. Figure shows the plot across all SHH‐MB patients in each data set [36, 37].

**Fig. S7**. REST‐dependent elevation of secreted proangiogenic molecules in MB cells. Secreted angiogenesis‐related protein molecules in conditioned medium of (A) D283 and (B) UW426‐R cells were done using an array kit (left panel). Densitometric analysis of angiogenic molecules is shown on the right (also see Table S1).

**Fig. S8**. REST and VEGFR1 expression in MB patients. (A‐B) IHC for VEGFR1 was performed on tumor sections from *Ptch* *^+/‐^* and *Ptch* *^+/‐^* */REST* *^TG^* transgenic mice, and in tumor‐bearing brain sections of PDOX mice to demonstrate vasculature changes in tumors. Arrowheads show the blood vessels. Scale bars: top (40×) = 10 μm, bottom (10×) = 20 μm. (C) *VEGFR1* mRNA expression profile in MB patient samples measured by microarray (GSE85217 [4], GSE37382 [37] and Pomeroy’ data sets [36]). Each dot corresponds to an individual patient. Data show individual variability and means ± SD. *P*‐values were obtained using the unpaired *t*‐test with Welch’s correction. ns, not significant. *P < 0.05, **P < 0.01, ***P < 0.001, ****P < 0.0001. (D) Scatter plot of correlation of *REST* mRNA expression and *VEGFR1* mRNA. Figure shows the plot across all SHH‐MB patients in each data set [36, 37].

**Fig. S9**. REST and ETS1 expression in MB patients. (A‐B) IHC for ETS1 was performed on tumor sections from *Ptch* *^+/‐^* and *Ptch* *^+/‐^* */REST* *^TG^* transgenic mice, and in tumor‐bearing brain sections of PDOX mice to demonstrate vasculature changes in tumors. Arrowheads show the blood vessels. Scale bars: top (40×) = 10 μm, bottom (10×) = 20 μm. (C) *ETS1* mRNA expression profile in MB patient samples measured by microarray (GSE85217 [4], GSE37382 [37]) and Pomeroy’ data sets [36]). Each dot corresponds to one individual patient. Data show individual variability and means ± SD. *P*‐values were obtained using the unpaired *t*‐test with Welch’s correction. ns, not significant. *P < 0.05, **P < 0.01, ***P < 0.001, ****P < 0.0001. (D) Scatter plot of correlation of *REST* mRNA expression and *ETS1* mRNA expression (GSE85217 [4], GSE37382 [37] and Pomeroy’ data sets [36]). Figure shows the plot across all SHH‐MB patients in each data set.

**Fig. S10.** *CD31, VEGFR1, ETS1* and *REST* mRNA expression in MB patient tumors. (A) Scatter plot of correlation of *REST* and *CD31* mRNA expression in SHH‐MBs (GSE85217 [4]). SHH‐subtype specific plots are shown. (B‐D) Profile of *CD31* mRNA expression in microarray data from WNT, Group3, and Group4 subgroup MB patient samples (left panel) (GSE85217 [4]). Each dot represents a patient. Data show individual variability and means ± SD. *P*‐values were obtained using the unpaired *t*‐test with Welch’s correction. ns, not significant. *P < 0.05, **P < 0.01, ***P < 0.001. Scatter plots show the correlation of *REST* mRNA expression and *CD31* mRNA expression. The second panel from the left shows data across all WNT‐ or Group3‐ or Group4‐MBs. Third, fourth and fifth panel show subtype specific correlative information for WNT‐ or Group3‐ or Group4‐MBs. (E‐H) VEGFR1 mRNA expression in MB patient tumors [4]. (I‐L) ETS1 mRNA expression in MB patient tumors [4].

**Table S1**. Densitometric analysis of proteomic array.

**Table S2.** List of angiogenesis‐related genes.

**Table S3.** List of differentially expressed genes between high‐REST and low‐REST patient tumors (volcano plot).

## Data Availability

The public data sets for gene expression analysis are available in GEO (https://www.ncbi.nlm.nih.gov/geo/) or R2: Genomics Analysis and Visualization Platform (https://hgserver1.amc.nl/cgi‐bin/r2/main.cgi).

## Data Availability

The RNA‐Seq data of MB cell lines described in this study has been deposited in NCBI Gene Expression Omnibus (GEO) with the accession number GSE164887.
