# Supplementary figures and images for: REST promotes ETS1‐dependent vascular growth in medulloblastoma
Source: Mol Oncol. 2021 Feb 7;15(5):1486–506. doi: 10.1002/1878-0261.12903 (PMC8096796; doi:10.1002/1878-0261.12903)

# Supplementary Figure 1

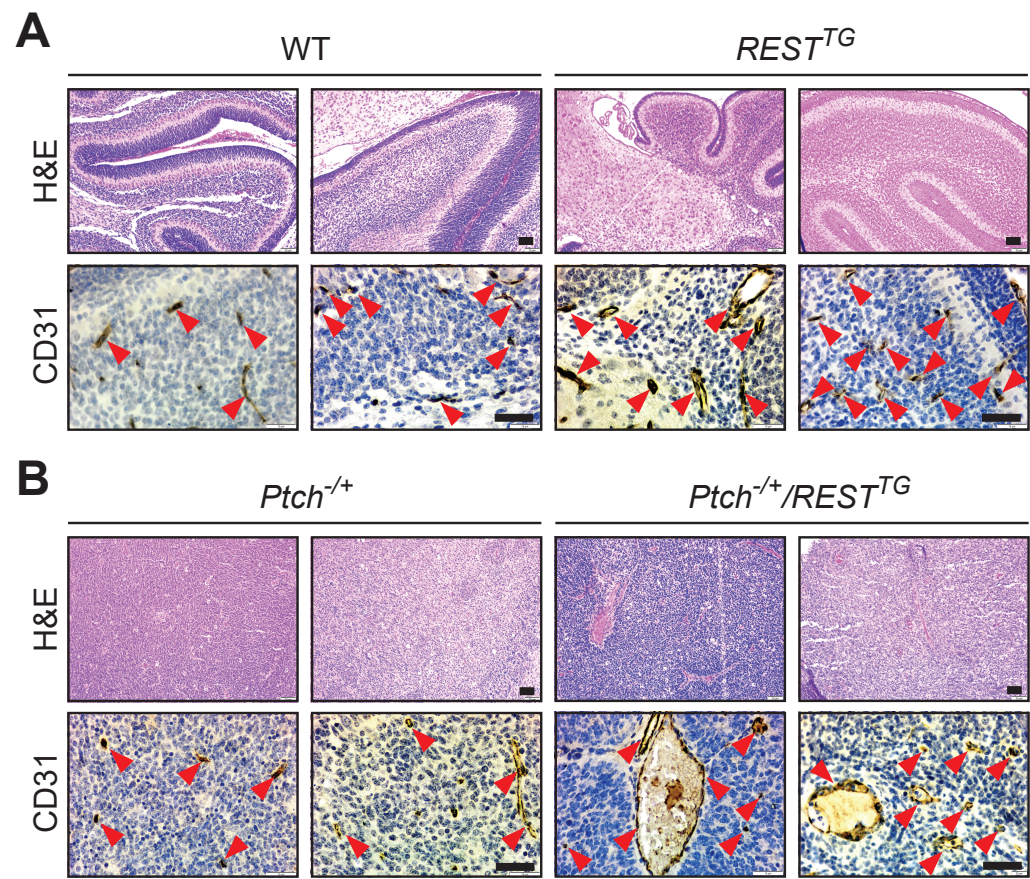

Supplement: Supplementary file 1 — Fig. S1. REST promotes vasculature in RESTTG cerebella and Ptch+/−/RESTTG. (A) Cerebellar sections from WT and REST TG mice and (B) tumor sections from Ptch +/‐ and Ptch +/‐ /REST TG transgenic mice to demonstrate the vasculature changes. Arrowheads show the blood vessels. (n=3). Scale bars; H&E=20 μm; CD31=10 μm. [file MOL2-15-1486-s013.pdf]

# Supplementary Figure 2

## G WNT markers

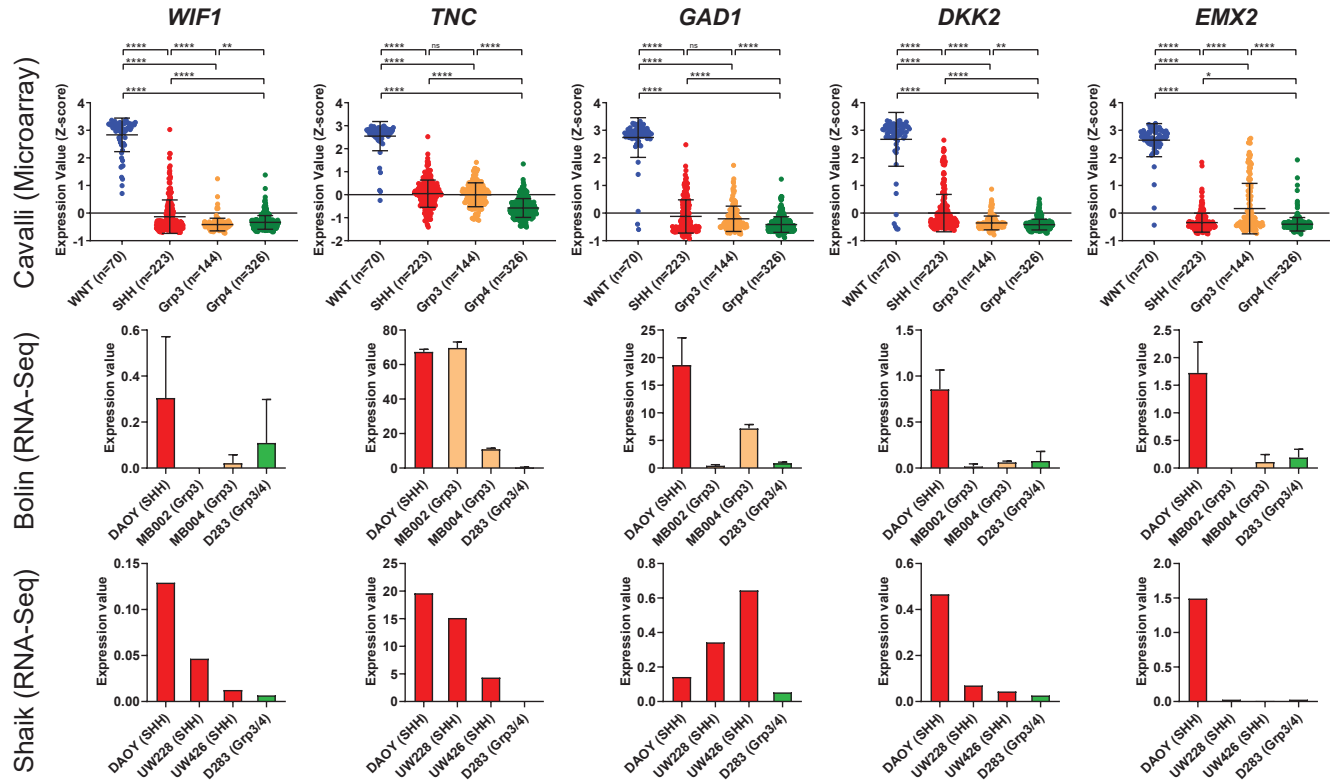

## H SHH markers

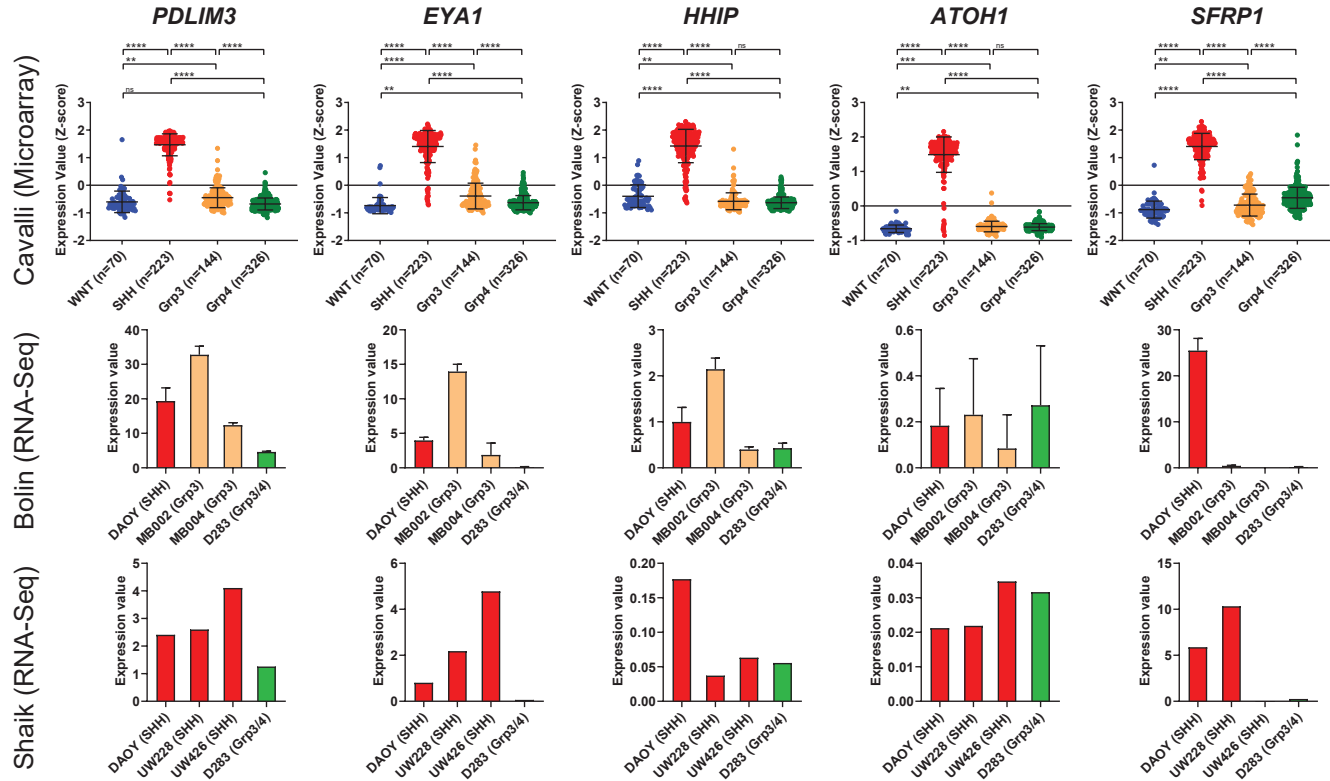

Supplement: Supplementary file 2 — Fig. S2. Gene expression profiles of subtype specific markers and hedgehog markers in MB cell lines and MB patients. (A) Unsupervised hierarchical cluster analysis of gene expression data using NanoString 22 genes in GSE86574. Expression values were Z‐score transformed. Red; high expression, blue; low expression. Arrowhead shows the position of DAOY clustered with MB_SHH patient samples. (B) Unsupervised hierarchical cluster analysis of gene expression data using NanoString 100 genes in GSE86574. (C) Unsupervised hierarchical cluster analysis of gene expression data using 33 hedgehog pathway related genes in publicly available microarray data [4]. (D) Unsupervised hierarchical cluster analysis of gene expression data using NanoString 22 genes [38] in GSE85217 [4]. (E) Unsupervised hierarchical cluster analysis of gene expression data using NanoString 22 genes in GSE107405 [35]. (F) Unsupervised hierarchical cluster analysis of gene expression data using NanoString 22 genes in our RNA‐seq data (Shaik). (G‐J) Gene expression profiles of subtype specific markers (NanoString 22 genes) (WNT, SHH, Group3 and Group4) in GSE85217 [4], GSE107405 [35] and our RNA‐seq data (Shaik). Data show individual variability and means ± SD. P‐values were obtained using the unpaired t‐test with Welch’s correction. ns, not significant. *P < 0.05, **P < 0.01, ***P < 0.001, ****P < 0.0001. [file MOL2-15-1486-s011.zip › mol212903-sup-0004-FigS2G-H.pdf]

# Supplementary Figure 2

I

Grp3 markers

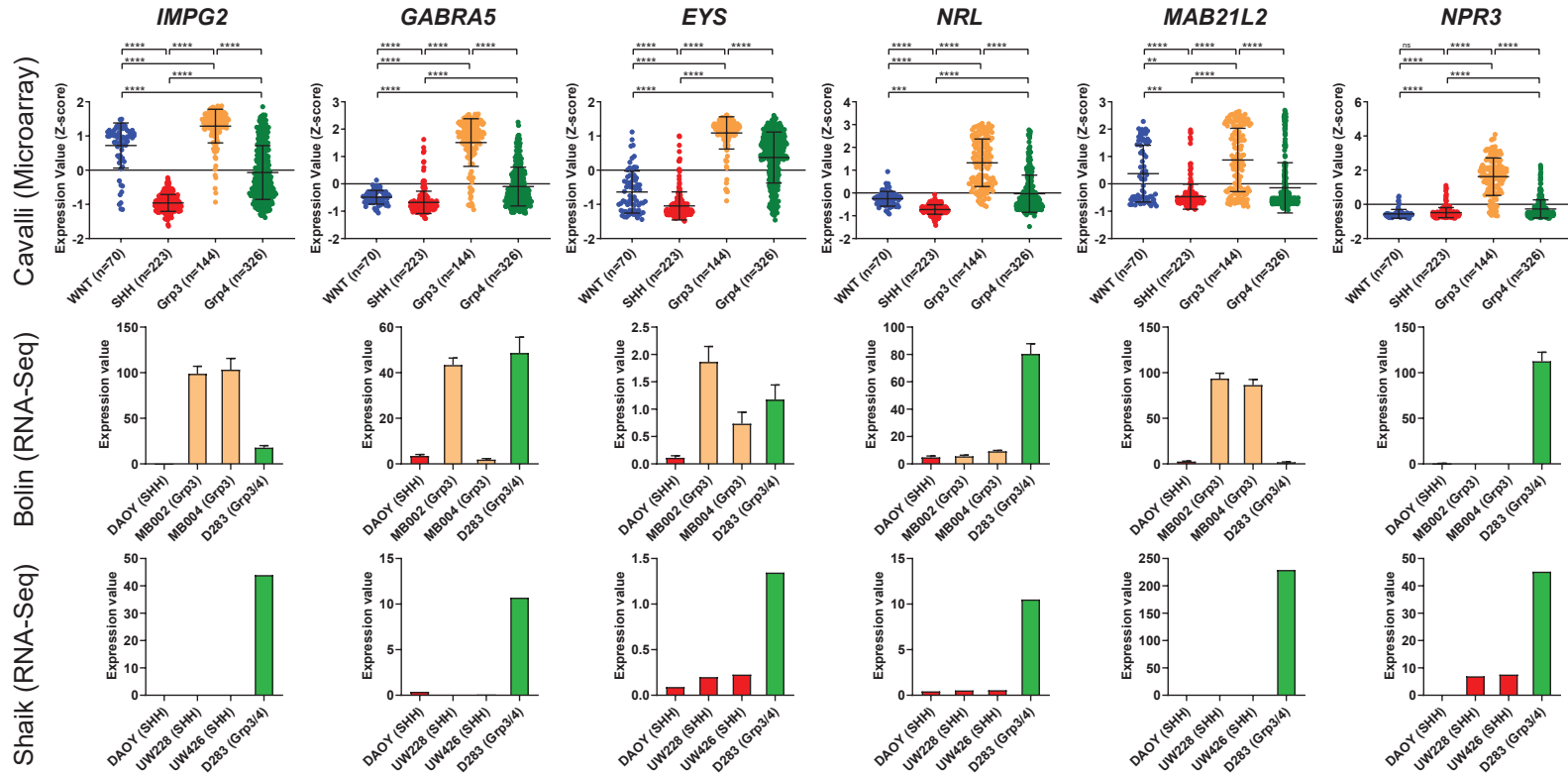

J

Grp4 markers

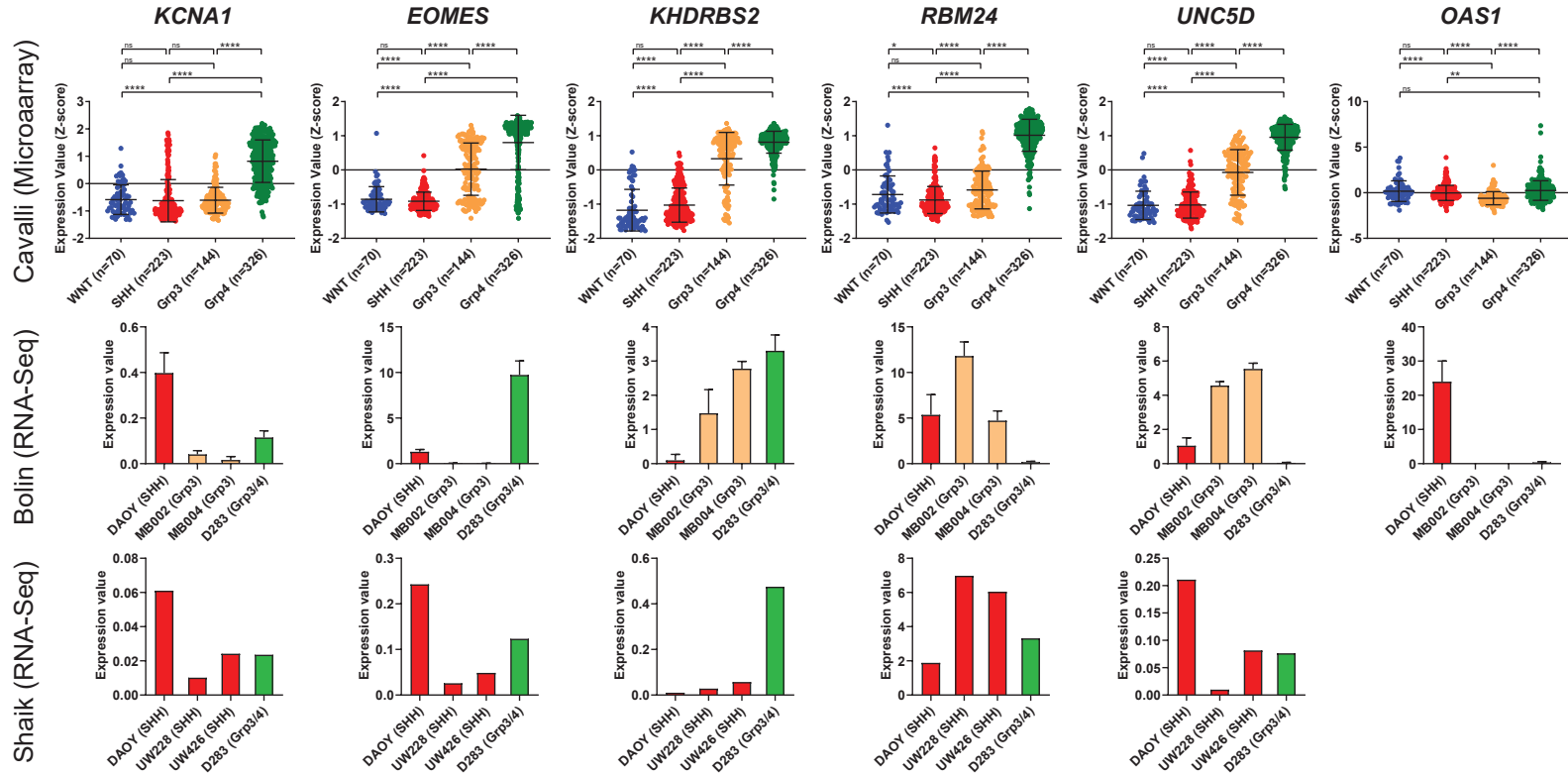

Supplement: Supplementary file 2 — Fig. S2. Gene expression profiles of subtype specific markers and hedgehog markers in MB cell lines and MB patients. (A) Unsupervised hierarchical cluster analysis of gene expression data using NanoString 22 genes in GSE86574. Expression values were Z‐score transformed. Red; high expression, blue; low expression. Arrowhead shows the position of DAOY clustered with MB_SHH patient samples. (B) Unsupervised hierarchical cluster analysis of gene expression data using NanoString 100 genes in GSE86574. (C) Unsupervised hierarchical cluster analysis of gene expression data using 33 hedgehog pathway related genes in publicly available microarray data [4]. (D) Unsupervised hierarchical cluster analysis of gene expression data using NanoString 22 genes [38] in GSE85217 [4]. (E) Unsupervised hierarchical cluster analysis of gene expression data using NanoString 22 genes in GSE107405 [35]. (F) Unsupervised hierarchical cluster analysis of gene expression data using NanoString 22 genes in our RNA‐seq data (Shaik). (G‐J) Gene expression profiles of subtype specific markers (NanoString 22 genes) (WNT, SHH, Group3 and Group4) in GSE85217 [4], GSE107405 [35] and our RNA‐seq data (Shaik). Data show individual variability and means ± SD. P‐values were obtained using the unpaired t‐test with Welch’s correction. ns, not significant. *P < 0.05, **P < 0.01, ***P < 0.001, ****P < 0.0001. [file MOL2-15-1486-s011.zip › mol212903-sup-0005-FigS2I-J.pdf]

## A Hippo signaling

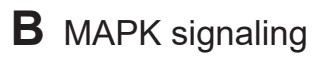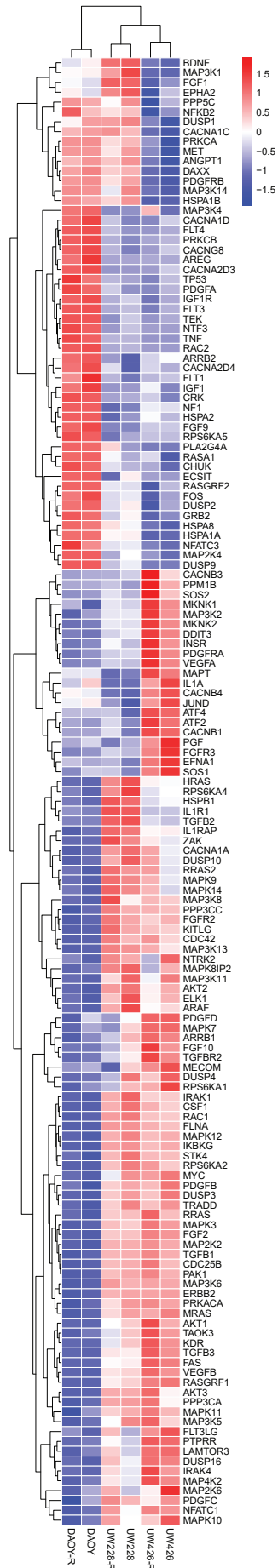

Supplement: Supplementary file 3 — Fig. S3. Heatmaps of differentially expressed genes in hippo signaling pathway and MAPK signaling pathway. [file MOL2-15-1486-s012.pdf]

Supplementary Figure 4

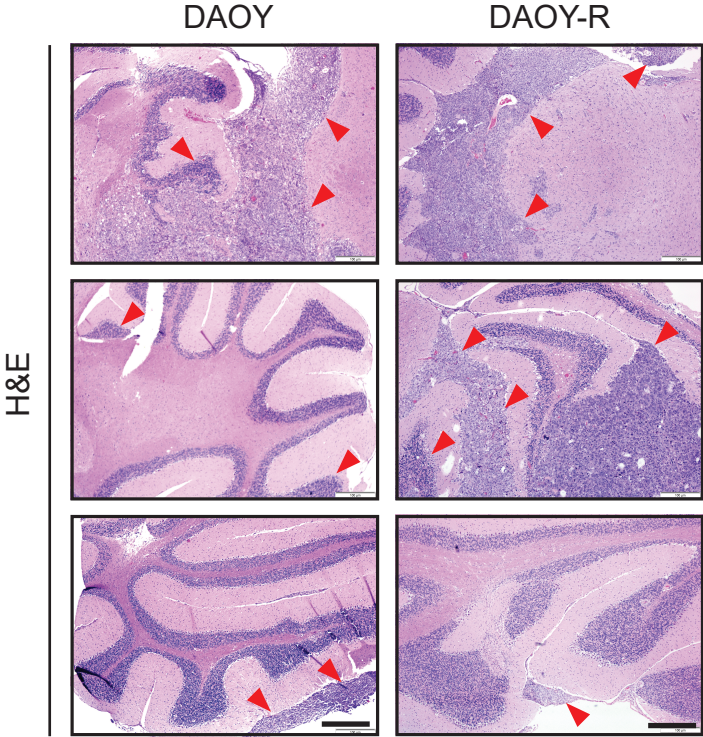

Supplement: Supplementary file 4 — Fig. S4. Human MBs with REST elevation have increased vasculature. H&E staining on brain sections from DAOY and DAOY‐REST (DAOY‐R) mice xenografts. n = 3. Scale bar; 100 μm. Arrowheads show tumor area. [file MOL2-15-1486-s003.pdf]

Supplementary Figure 5

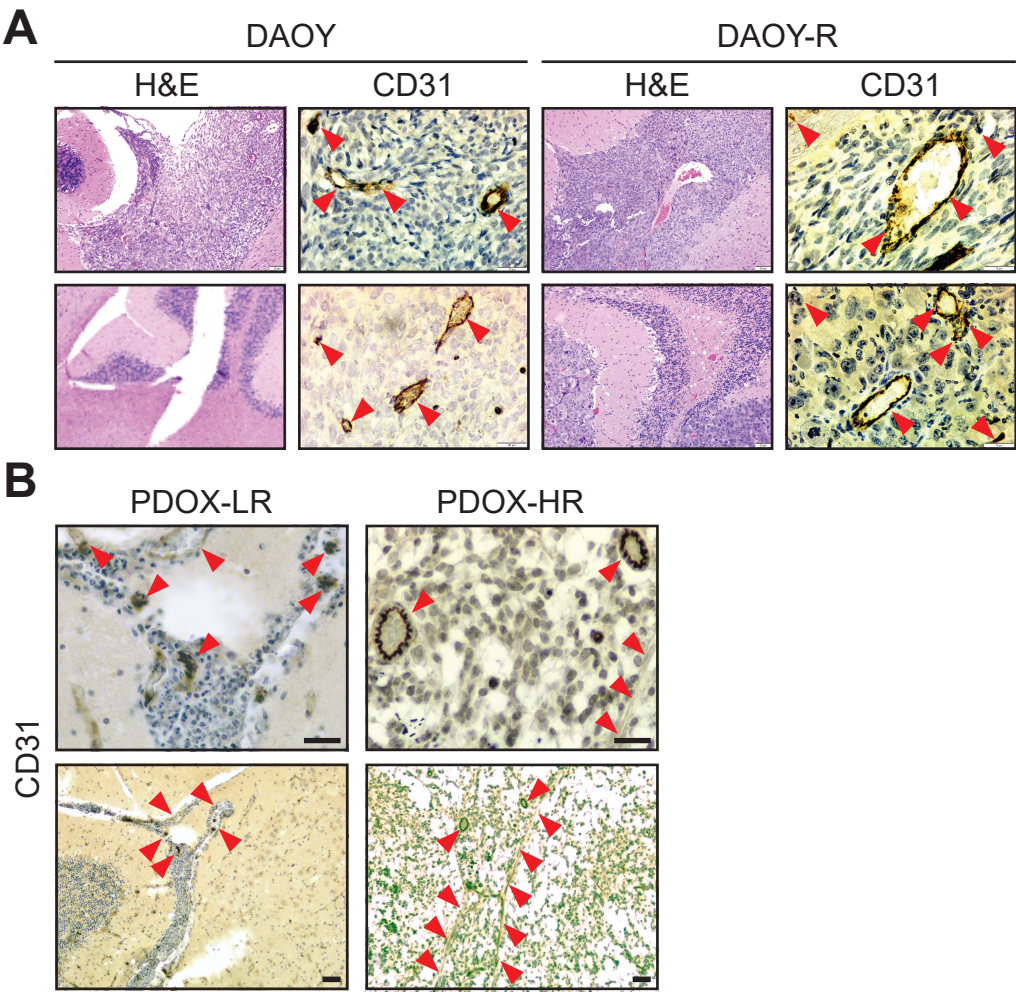

Supplement: Supplementary file 5 — Fig. S5. REST promotes vasculature in DAOY‐R and PDOX‐HR tumors. (A) DAOY/DAOY‐R tumors and (B) PDOX‐LR and PDOX‐HR tumors to demonstrate the vasculature changes. Arrowheads show the blood vessels. (n = 3). Scale bars in A; H&E = 20 μm; CD31 = 10 μm. Scale bars in B: top (40×) = 10 μm, bottom (10×) = 20 μm. [file MOL2-15-1486-s008.pdf]

**A**

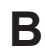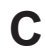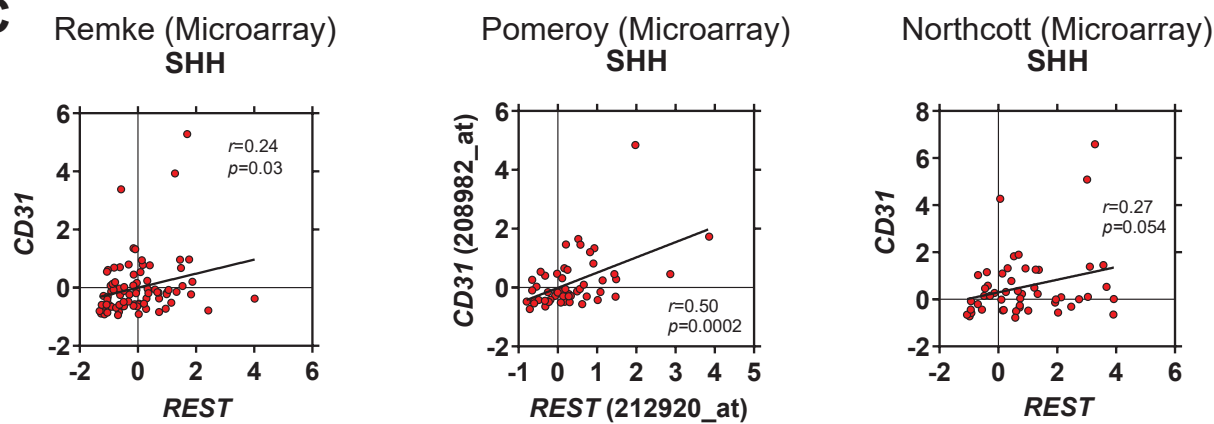

Supplement: Supplementary file 6 — Fig. S6. REST and CD31 expression in MB patients. (A) REST mRNA expression profile in MB patient samples measured by microarray (GSE85217 [4], GSE37382 [37] and Pomeroy’ data sets [36]). Each dot corresponds to one individual patient. Data show individual variability and means ± SD. P‐values were obtained using the unpaired t‐test with Welch’s correction. ns, not significant. *P < 0.05, **P < 0.01, ***P < 0.001, ****P < 0.0001. (B) CD31 mRNA expression profile in MB patient samples measured by microarray (GSE85217 [4], GSE37382 [37] and Pomeroy’ data sets [36]). Each dot corresponds to one individual patient. Data show individual variability and means ± SD. P‐values were obtained using the unpaired t‐test with Welch’s correction. ns, not significant. *P < 0.05, **P < 0.01, ***P < 0.001, ****P < 0.0001. (C) Scatter plot of correlation of REST mRNA expression and CD31 mRNA. Figure shows the plot across all SHH‐MB patients in each data set [36, 37]. [file MOL2-15-1486-s005.pdf]

Supplementary Figure 7

A

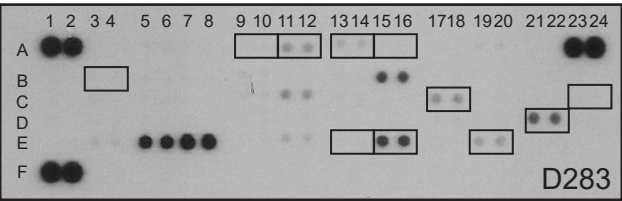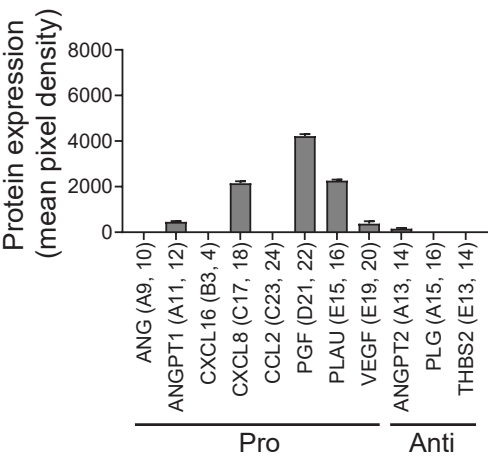

B

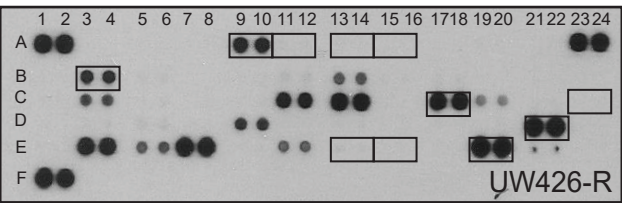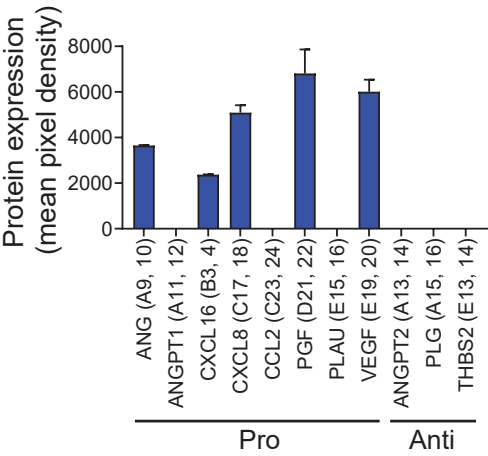

Supplement: Supplementary file 7 — Fig. S7. REST‐dependent elevation of secreted proangiogenic molecules in MB cells. Secreted angiogenesis‐related protein molecules in conditioned medium of (A) D283 and (B) UW426‐R cells were done using an array kit (left panel). Densitometric analysis of angiogenic molecules is shown on the right (also see Table S1). [file MOL2-15-1486-s010.pdf]

Supplementary Figure 8

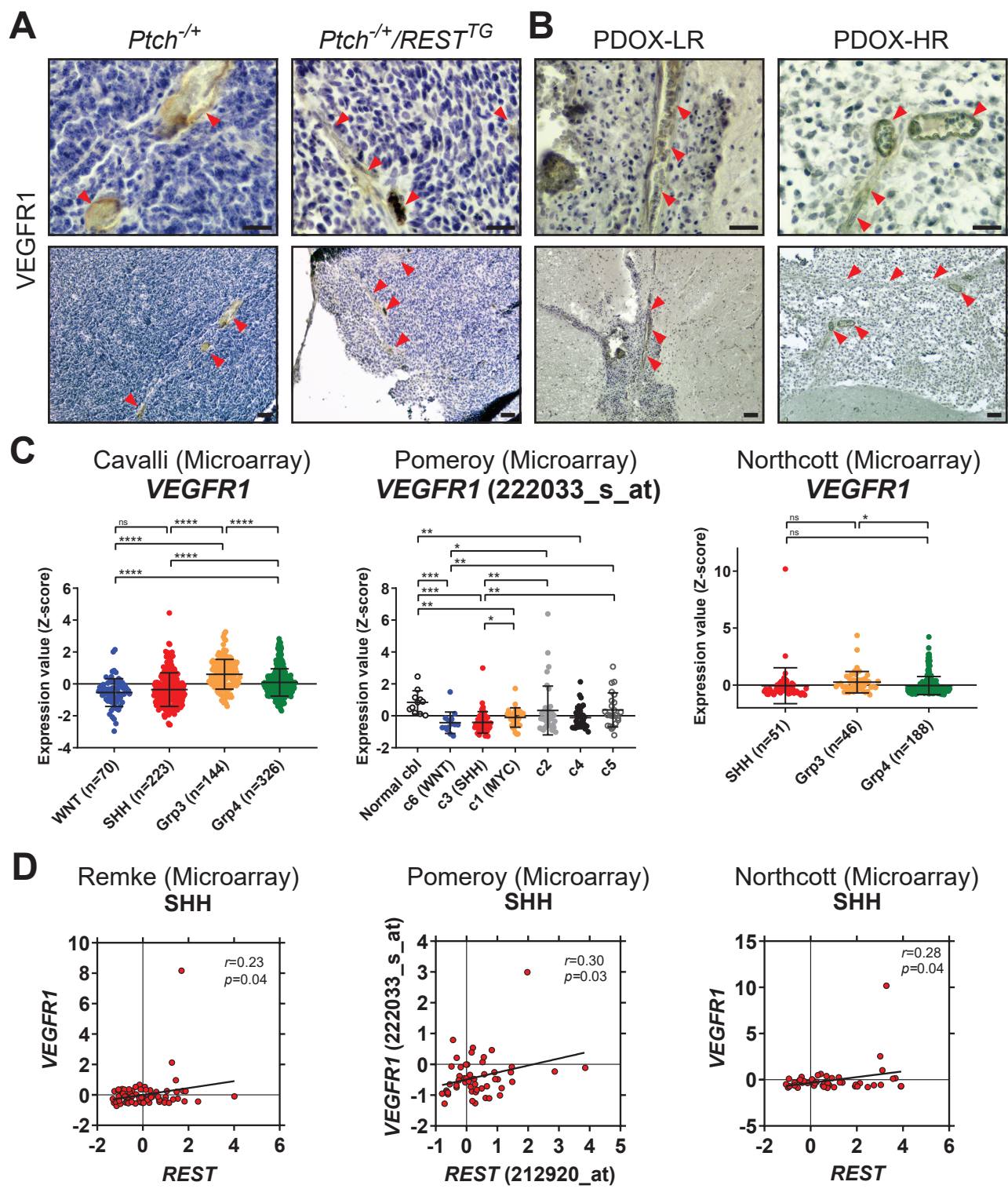

Supplement: Supplementary file 8 — Fig. S8. REST and VEGFR1 expression in MB patients. (A‐B) IHC for VEGFR1 was performed on tumor sections from Ptch +/‐ and Ptch +/‐ /REST TG transgenic mice, and in tumor‐bearing brain sections of PDOX mice to demonstrate vasculature changes in tumors. Arrowheads show the blood vessels. Scale bars: top (40×) = 10 μm, bottom (10×) = 20 μm. (C) VEGFR1 mRNA expression profile in MB patient samples measured by microarray (GSE85217 [4], GSE37382 [37] and Pomeroy’ data sets [36]). Each dot corresponds to an individual patient. Data show individual variability and means ± SD. P‐values were obtained using the unpaired t‐test with Welch’s correction. ns, not significant. *P < 0.05, **P < 0.01, ***P < 0.001, ****P < 0.0001. (D) Scatter plot of correlation of REST mRNA expression and VEGFR1 mRNA. Figure shows the plot across all SHH‐MB patients in each data set [36, 37]. [file MOL2-15-1486-s009.pdf]

# Supplementary Figure 9

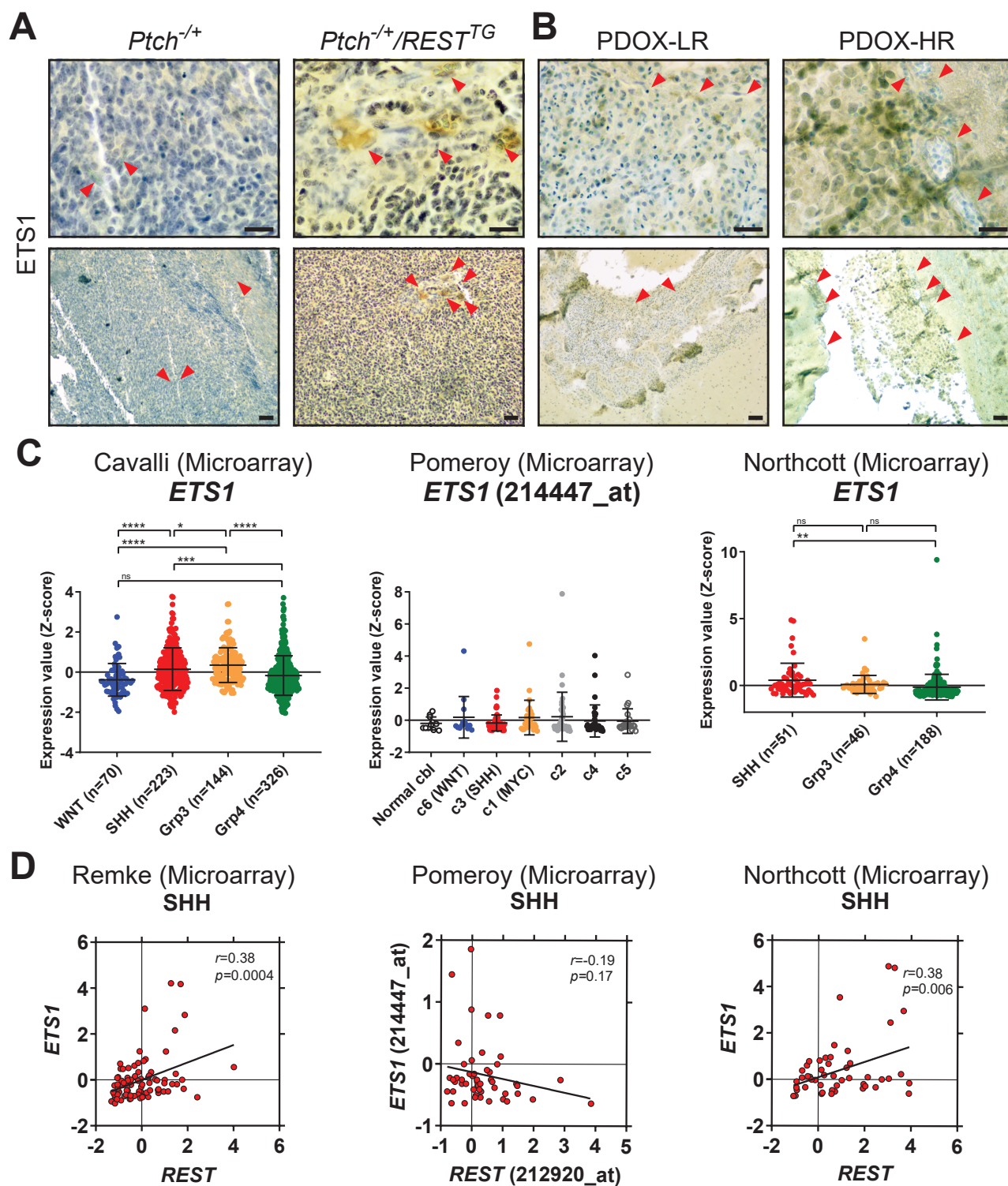

Supplement: Supplementary file 9 — Fig. S9. REST and ETS1 expression in MB patients. (A‐B) IHC for ETS1 was performed on tumor sections from Ptch +/‐ and Ptch +/‐ /REST TG transgenic mice, and in tumor‐bearing brain sections of PDOX mice to demonstrate vasculature changes in tumors. Arrowheads show the blood vessels. Scale bars: top (40×) = 10 μm, bottom (10×) = 20 μm. (C) ETS1 mRNA expression profile in MB patient samples measured by microarray (GSE85217 [4], GSE37382 [37]) and Pomeroy’ data sets [36]). Each dot corresponds to one individual patient. Data show individual variability and means ± SD. P‐values were obtained using the unpaired t‐test with Welch’s correction. ns, not significant. *P < 0.05, **P < 0.01, ***P < 0.001, ****P < 0.0001. (D) Scatter plot of correlation of REST mRNA expression and ETS1 mRNA expression (GSE85217 [4], GSE37382 [37] and Pomeroy’ data sets [36]). Figure shows the plot across all SHH‐MB patients in each data set. [file MOL2-15-1486-s002.pdf]

# Supplementary Figure 10

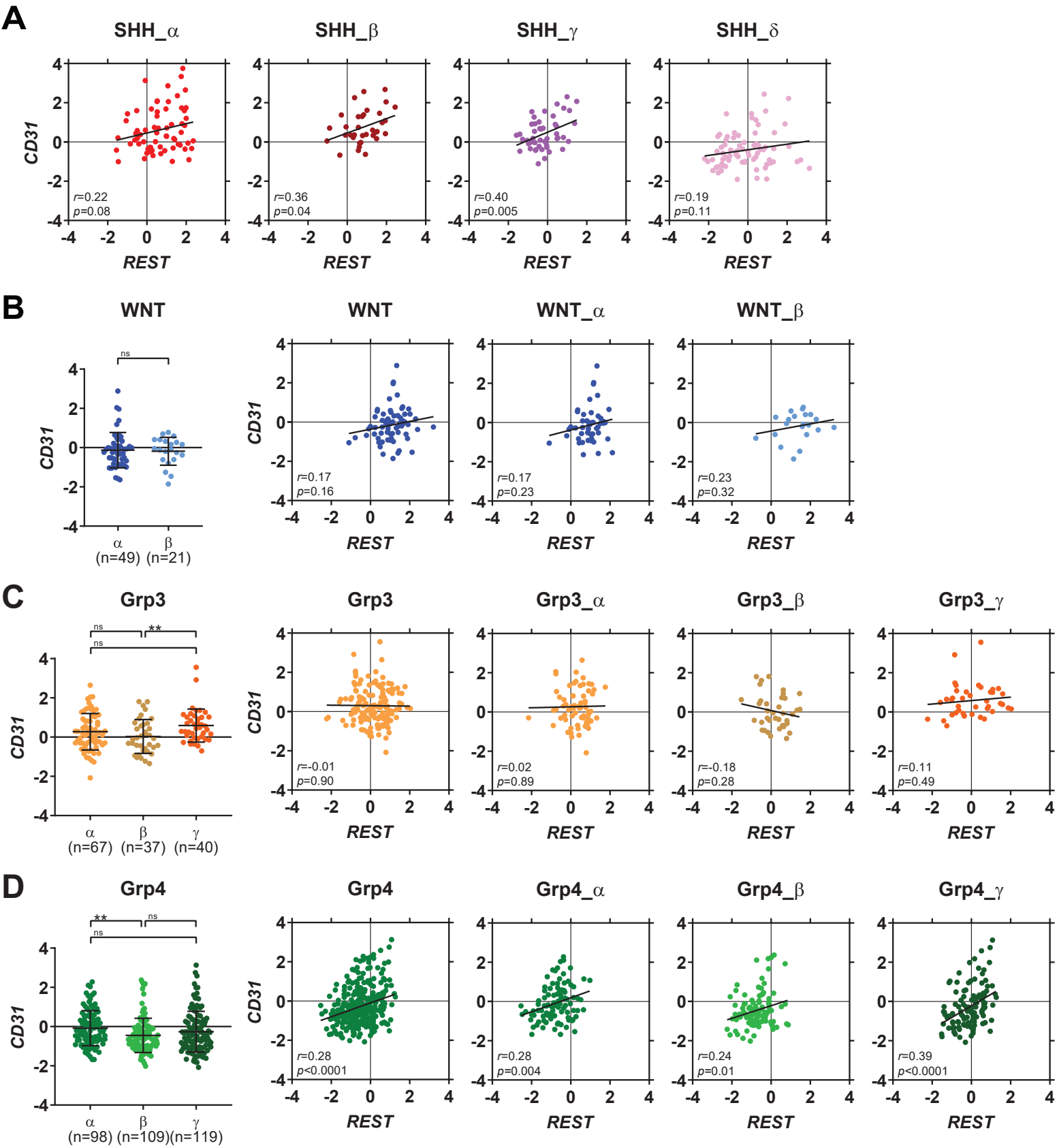

Supplement: Supplementary file 10 — Fig. S10. CD31, VEGFR1, ETS1 and REST mRNA expression in MB patient tumors. (A) Scatter plot of correlation of REST and CD31 mRNA expression in SHH‐MBs (GSE85217 [4]). SHH‐subtype specific plots are shown. (B‐D) Profile of CD31 mRNA expression in microarray data from WNT, Group3, and Group4 subgroup MB patient samples (left panel) (GSE85217 [4]). Each dot represents a patient. Data show individual variability and means ± SD. P‐values were obtained using the unpaired t‐test with Welch’s correction. ns, not significant. *P < 0.05, **P < 0.01, ***P < 0.001. Scatter plots show the correlation of REST mRNA expression and CD31 mRNA expression. The second panel from the left shows data across all WNT‐ or Group3‐ or Group4‐MBs. Third, fourth and fifth panel show subtype specific correlative information for WNT‐ or Group3‐ or Group4‐MBs. (E‐H) VEGFR1 mRNA expression in MB patient tumors [4]. (I‐L) ETS1 mRNA expression in MB patient tumors [4]. [file MOL2-15-1486-s006.zip › mol212903-sup-0016-FigS10A-d.pdf]

Supplementary Figure 10

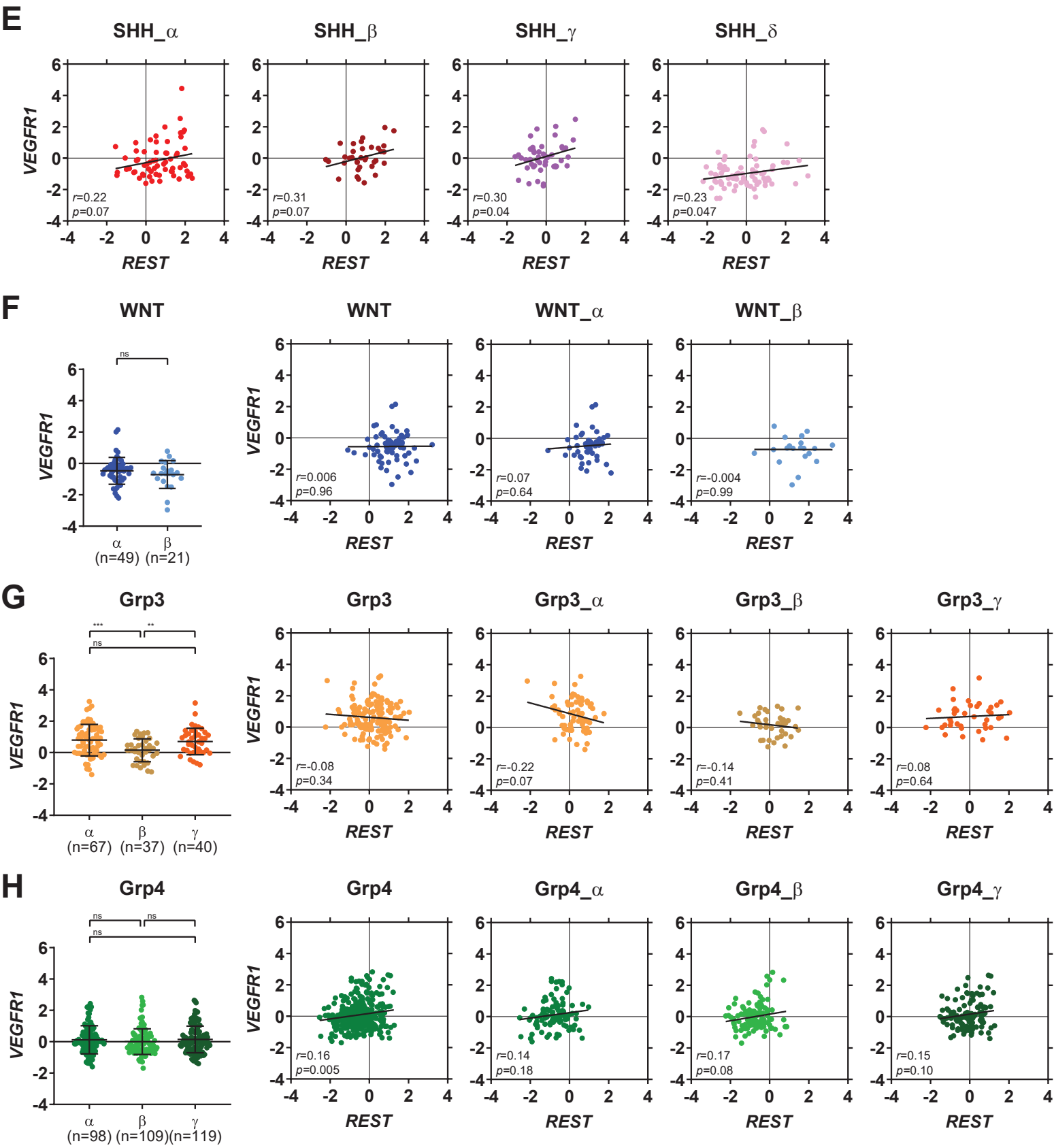

Supplement: Supplementary file 10 — Fig. S10. CD31, VEGFR1, ETS1 and REST mRNA expression in MB patient tumors. (A) Scatter plot of correlation of REST and CD31 mRNA expression in SHH‐MBs (GSE85217 [4]). SHH‐subtype specific plots are shown. (B‐D) Profile of CD31 mRNA expression in microarray data from WNT, Group3, and Group4 subgroup MB patient samples (left panel) (GSE85217 [4]). Each dot represents a patient. Data show individual variability and means ± SD. P‐values were obtained using the unpaired t‐test with Welch’s correction. ns, not significant. *P < 0.05, **P < 0.01, ***P < 0.001. Scatter plots show the correlation of REST mRNA expression and CD31 mRNA expression. The second panel from the left shows data across all WNT‐ or Group3‐ or Group4‐MBs. Third, fourth and fifth panel show subtype specific correlative information for WNT‐ or Group3‐ or Group4‐MBs. (E‐H) VEGFR1 mRNA expression in MB patient tumors [4]. (I‐L) ETS1 mRNA expression in MB patient tumors [4]. [file MOL2-15-1486-s006.zip › mol212903-sup-0017-FigS10E-H.pdf]

Supplementary Figure 10

I

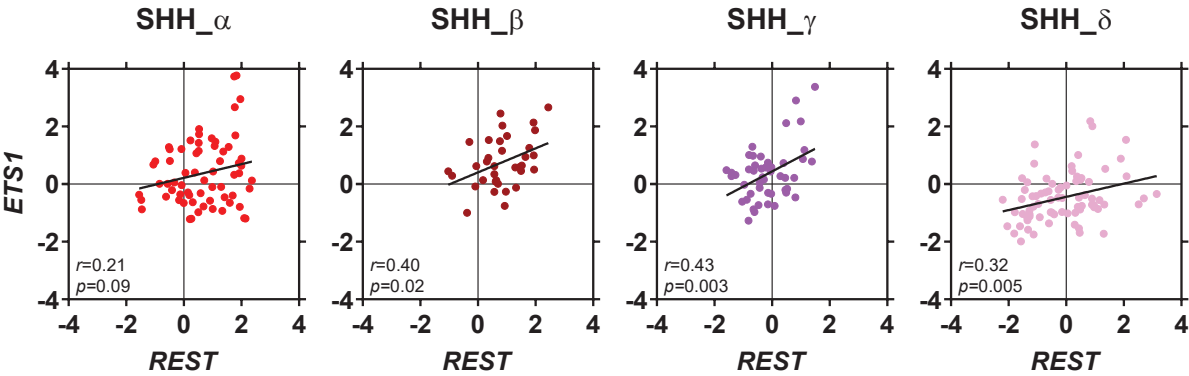

J

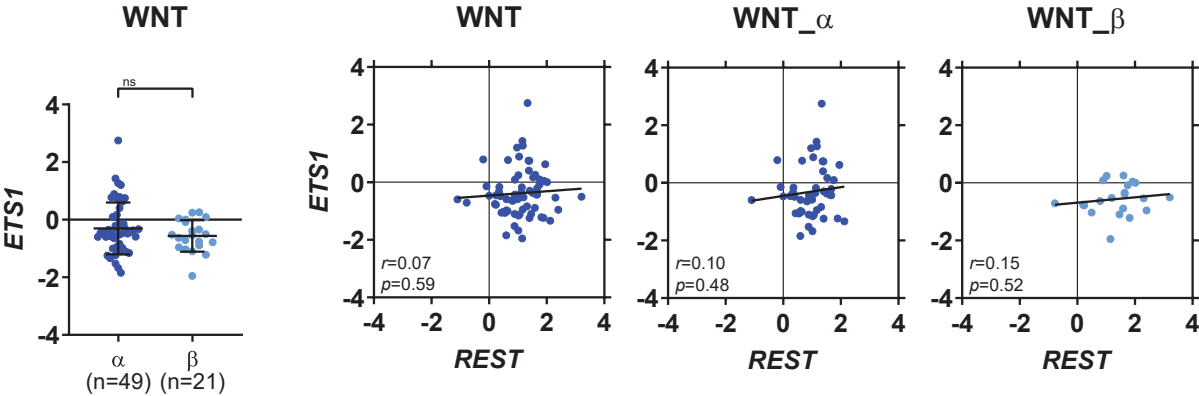

K

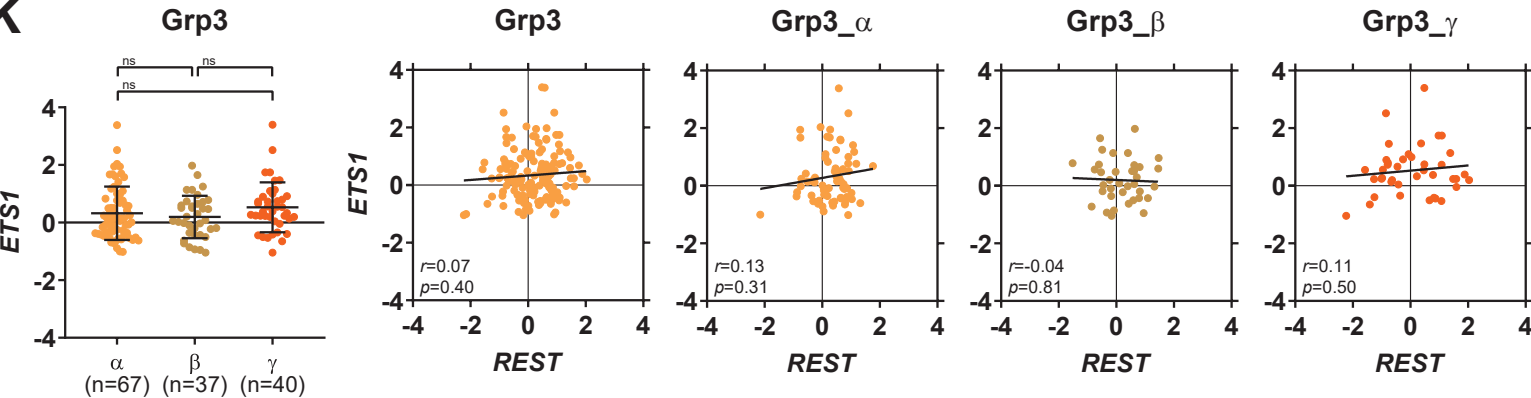

L

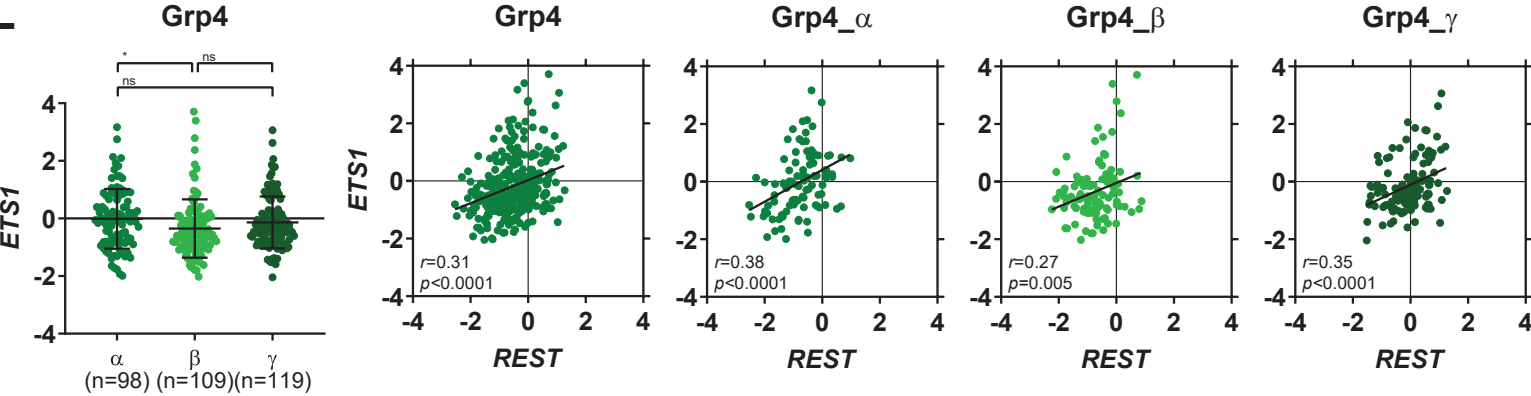

Supplement: Supplementary file 10 — Fig. S10. CD31, VEGFR1, ETS1 and REST mRNA expression in MB patient tumors. (A) Scatter plot of correlation of REST and CD31 mRNA expression in SHH‐MBs (GSE85217 [4]). SHH‐subtype specific plots are shown. (B‐D) Profile of CD31 mRNA expression in microarray data from WNT, Group3, and Group4 subgroup MB patient samples (left panel) (GSE85217 [4]). Each dot represents a patient. Data show individual variability and means ± SD. P‐values were obtained using the unpaired t‐test with Welch’s correction. ns, not significant. *P < 0.05, **P < 0.01, ***P < 0.001. Scatter plots show the correlation of REST mRNA expression and CD31 mRNA expression. The second panel from the left shows data across all WNT‐ or Group3‐ or Group4‐MBs. Third, fourth and fifth panel show subtype specific correlative information for WNT‐ or Group3‐ or Group4‐MBs. (E‐H) VEGFR1 mRNA expression in MB patient tumors [4]. (I‐L) ETS1 mRNA expression in MB patient tumors [4]. [file MOL2-15-1486-s006.zip › mol212903-sup-0018-FigS10I-l.pdf]
