# Supplementary material for: REST promotes ETS1‐dependent vascular growth in medulloblastoma
Source: Mol Oncol. 2021 Feb 7;15(5):1486–506. doi: 10.1002/1878-0261.12903 (PMC8096796; doi:10.1002/1878-0261.12903)

Supplementary Figure 2

C Hedgehog pathway related genes; Cavalli (Microarray)

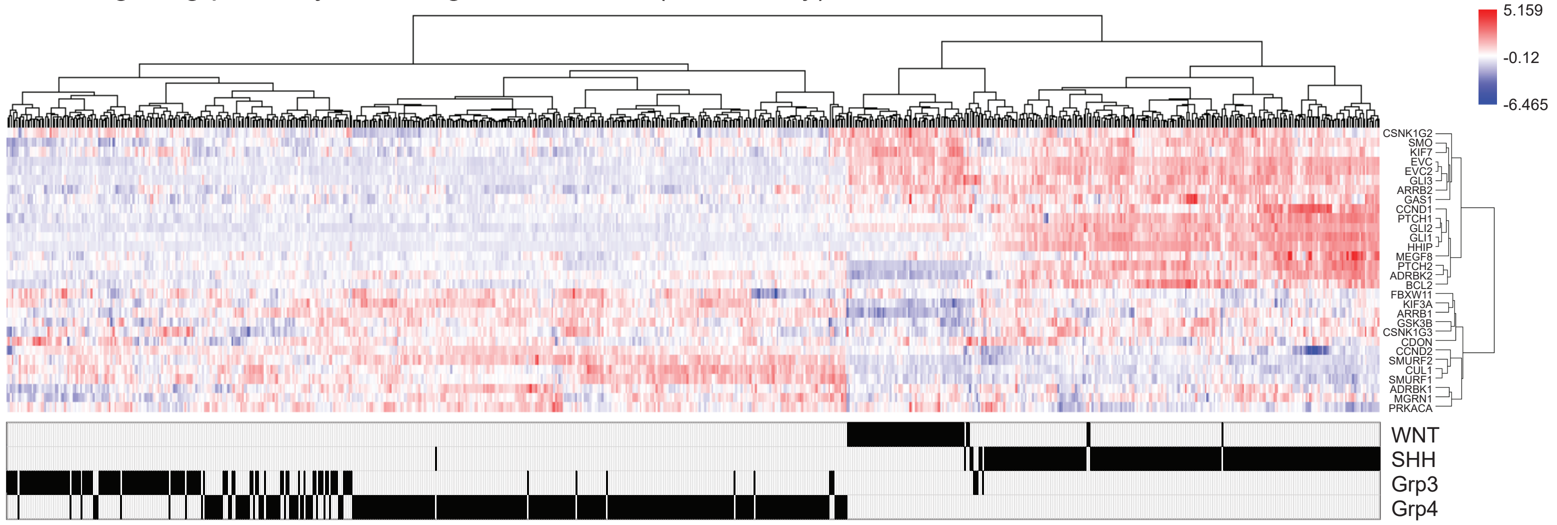

D NanoString 22 genes; Cavalli (Microarray)

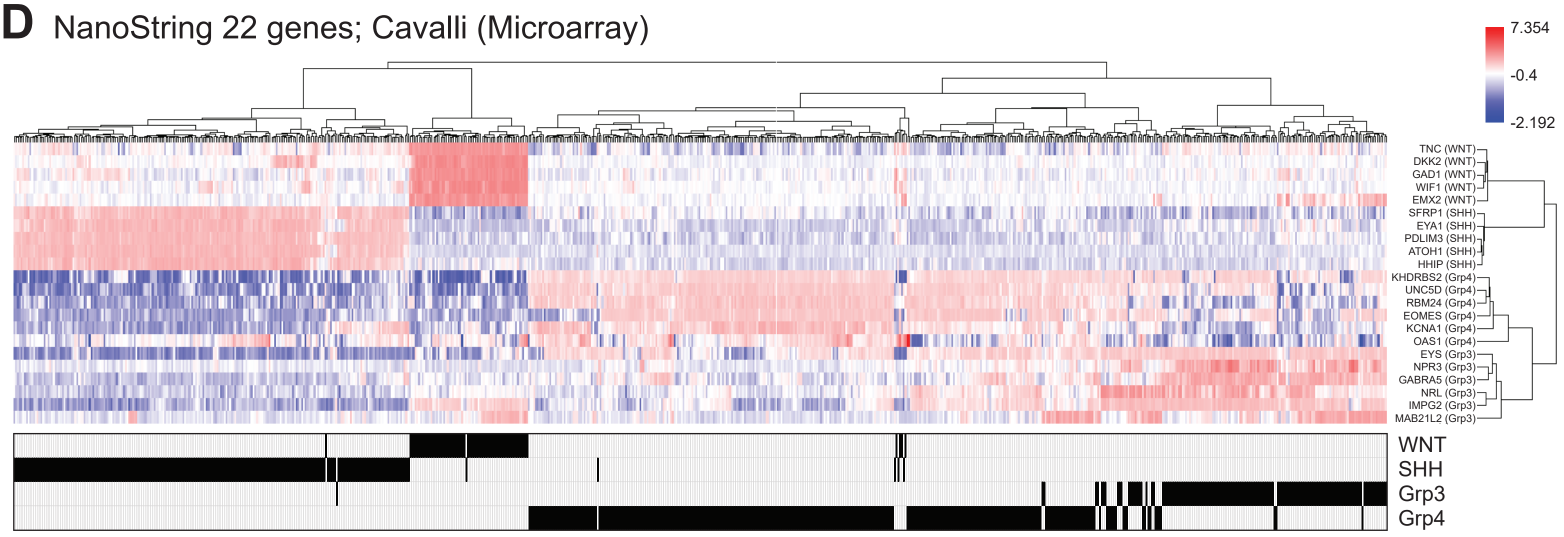

E NanoString 22 genes; Bolin (RNA-Seq)

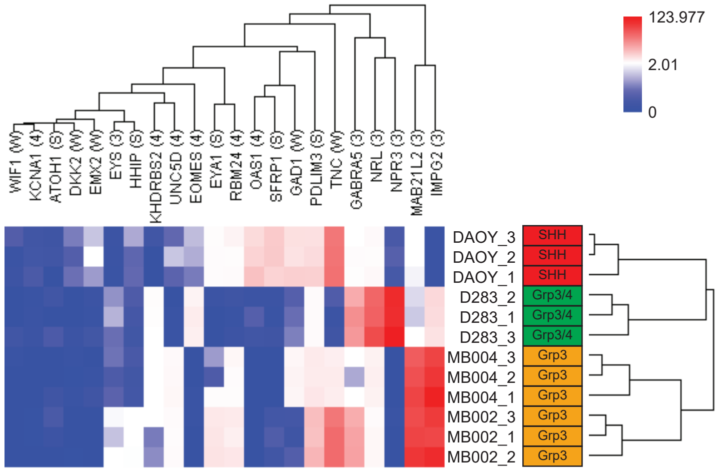

F NanoString 22 genes; Shaik (RNA-Seq)

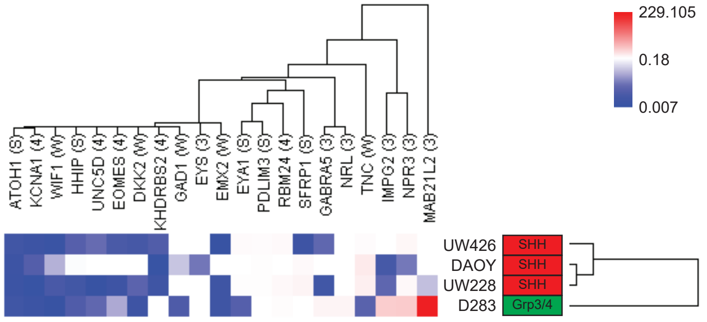

Supplement: Supplementary file 2 — Fig. S2. Gene expression profiles of subtype specific markers and hedgehog markers in MB cell lines and MB patients. (A) Unsupervised hierarchical cluster analysis of gene expression data using NanoString 22 genes in GSE86574. Expression values were Z‐score transformed. Red; high expression, blue; low expression. Arrowhead shows the position of DAOY clustered with MB_SHH patient samples. (B) Unsupervised hierarchical cluster analysis of gene expression data using NanoString 100 genes in GSE86574. (C) Unsupervised hierarchical cluster analysis of gene expression data using 33 hedgehog pathway related genes in publicly available microarray data [4]. (D) Unsupervised hierarchical cluster analysis of gene expression data using NanoString 22 genes [38] in GSE85217 [4]. (E) Unsupervised hierarchical cluster analysis of gene expression data using NanoString 22 genes in GSE107405 [35]. (F) Unsupervised hierarchical cluster analysis of gene expression data using NanoString 22 genes in our RNA‐seq data (Shaik). (G‐J) Gene expression profiles of subtype specific markers (NanoString 22 genes) (WNT, SHH, Group3 and Group4) in GSE85217 [4], GSE107405 [35] and our RNA‐seq data (Shaik). Data show individual variability and means ± SD. P‐values were obtained using the unpaired t‐test with Welch’s correction. ns, not significant. *P < 0.05, **P < 0.01, ***P < 0.001, ****P < 0.0001. [file MOL2-15-1486-s011.zip › mol212903-sup-0003-FigS2C-F.pdf]
