# Supplementary material for: REST promotes ETS1‐dependent vascular growth in medulloblastoma
Source: Mol Oncol. 2021 Feb 7;15(5):1486–506. doi: 10.1002/1878-0261.12903 (PMC8096796; doi:10.1002/1878-0261.12903)
Supplement: Supplementary file 11 — Table S1. Densitometric analysis of proteomic array. [file MOL2-15-1486-s001.pdf]

Supplementary Table 1; Densitometric analysis of proteomic array.

Gene name			Position	UW228		UW426		UW426-REST		DAOY		D283	
				SHH		SHH		SHH		SHH		Group3/4	
Reference spots			A1, A2	7336	6771	5905	5464	5660	5267	5135	4920	7896	6389
INHBA	*Activin A*		A5, A6	433	378	0	0	0	0	5638	5334	0	0
ADAMTS1	*ADAMTS-1*		A7, A8	0	0	0	0	0	0	0	0	0	0
ANG	*Angiogenin*		A9, A10	360	333	348	515	3637	3663	4869	4490	0	0
ANGPT1	*Angiopoietin-1*	*Ang-1*	A11, A12	200	227	0	0	0	0	2059	2237	481	426
ANGPT2	*Angiopoietin-2*	*Ang-2*	A13, A14	58	82	0	0	0	0	18	92	182	130
PLG	*Angiostatin/Plasminogen*		A15, A16	0	0	0	0	0	0	0	0	0	0
AREG	*Amphiregulin*	*AR*	A17, A18	0	0	0	0	0	0	0	0	0	0
ARTN	*Artemin*		A19, A20	0	0	0	0	0	0	0	0	0	0
Reference spots			A23, A24	8520	7011	7372	5781	6547	6950	5606	5418	7537	7406
F3	*Coagulation Factor III*	*TF*	B1, B2	545	530	0	0	0	0	0	0	0	0
CXCL16			B3, B4	275	280	2226	2126	2379	2359	1916	2307	0	0
DPP4	*DPPIV*	*CD26*	B5, B6	430	458	0	0	0	0	0	0	0	0
EGF			B7, B8	0	0	0	0	0	0	0	0	0	0
PROK1	*EG-VEGF*	*PK1*	B9, B10	0	0	0	0	0	0	0	0	0	0
ENG	*Endoglin*	*CD105*	B11, B12	0	0	0	0	0	0	0	0	0	0
COL18A1	*Endostatin/Collagen XVIII*		B13, B14	415	473	3585	3690	3602	3938	867	902	0	0
EDN1	*Endothelin-1*	*ET-1*	B15, B16	623	575	0	0	0	0	0	0	3673	3645
FGF1	*FGF acidic*	*FGF-1*	B17, B18	0	0	0	0	0	0	0	0	0	0
FGF2	*FGF basic*	*FGF2*	B19, B20	0	0	0	0	0	0	0	0	0	0
FGF4	*FGF-4*		B21, B22	0	0	0	0	0	0	0	0	0	0
FGF7	*FGF-7*	*KGF*	B23, B24	0	0	0	0	0	0	0	0	0	0
GDNF			C1, C2	0	0	0	0	0	0	0	0	0	0
CSF2	*GM-CSF*		C3, C4	4321	4005	2109	1874	1875	1743	2269	2598	0	0
HBEGF	*HB-EGF*		C5, C6	0	0	0	0	0	0	0	0	0	0
HGF			C7, C8	0	0	0	0	0	0	0	0	0	0
IGFBP1	*IGFBP-1*		C9, C10	0	0	0	0	0	0	0	0	0	0
IGFBP2	*IGFBP-2*		C11, C12	0	0	2949	3010	4589	4462	2361	2247	2402	2041
IGFBP3	*IGFBP-3*		C13, C14	4984	5054	4390	4288	6184	5746	4822	4939	0	0
IL1B	*IL-1beta*	*IL-1F2*	C15, C16	0	0	0	0	0	0	0	0	0	0
CXCL8	*IL-8*		C17, C18	6125	5637	6280	5508	5318	4856	5643	5061	2213	2092
TGFB1	*LAP (TGF-b1)*		C19, C20	0	0	0	0	988	823	539	358	0	0
LEP	*Leptin*		C21, C22	0	0	0	0	0	0	0	0	0	0
CCL2	*MCP-1*		C23, C24	2264	1696	0	0	0	0	5955	5363	0	0
CCL3	*MIP-1a*		D1, D2	0	0	0	0	0	0	0	0	0	0
MMP8	*MMP-8*		D3, D4	0	0	0	0	0	0	0	0	0	0
MMP9	*MMP-9*		D5, D6	0	0	0	0	0	0	1197	1283	0	0
NRG1	*NRG1-beta1*	*HRG1-b1*	D7, D8	0	0	0	0	0	0	0	0	0	0
PTX3	*Pentraxin 3 (PTX3)*	*TSG-14*	D9, D10	2282	2099	1792	1762	2405	2476	2999	2986	0	0
TYMP	*PD-ECGF*		D11, D12	0	0	0	0	0	0	0	0	0	0
PDGFA	*PDGF-AA*		D13, D14	0	0	5165	4702	0	0	4733	4547	0	0
PDGFB	*PDGF-AB/PDGF-BB*		D15, D16	0	0	0	0	0	0	0	0	0	0
PSPN	*Persephin*		D17, D18	0	0	0	0	0	0	0	0	0	0
PF4	*Platelet factor 4 (PF4)*	*CXCL4*	D19, D20	0	0	0	0	0	0	0	0	0	0
PGF	*PLGF*		D21, D22	774	1064	3789	3379	6070	7553	2778	2633	4158	4280
PRL	*Prolactin*		D23, D24	0	0	0	0	0	0	0	0	0	0
SERPINB5	*Serpin B5*	*Maspin*	E1, E2	0	0	0	0	0	0	0	0	0	0
SERPINE1	*Serpin E1*	*PAI-1*	E3, E4	5053	3584	4417	4072	4406	4373	2269	2379	0	0
SERPINF1	*SerpinF1*	*PEDF*	E5, E6	0	0	978	1234	1438	1621	0	0	4889	4651
TIMP1	*TIMP-1*		E7, E8	7182	6235	6249	6491	5361	4889	6153	5361	5660	5134
TIMP4	*TIMP-4*		E9, E10	0	0	0	0	0	0	2400	2117	0	0
THBS1	*Thrombospondin-1*	*TSP-1*	E11, E12	2615	2395	1177	1185	1469	1347	2376	2236	218	219
THBS2	*Thrombospondin-2*	*TSP-2*	E13, E14	0	0	0	0	0	0	0	0	0	0
PLAU	*uPA*		E15, E16	6393	5297	0	0	0	0	6568	5264	2298	2232
VASH1	*Vasohibin*		E17, E18	0	0	0	0	0	0	0	0	0	0
VEGF			E19, E20	2360	2469	5911	5152	6381	5634	3196	2964	305	453
VEGFC	*VEGF-C*		E21, E22	0	0	0	0	0	0	0	0	0	0
Reference spots			F1, F2	6740	6780	5950	5091	5567	5432	5583	5688	6981	6777
Negative Control			F23, F24	0	0	0	0	0	0	0	0	0	0
