# Supplementary material for: REST promotes ETS1‐dependent vascular growth in medulloblastoma
Source: Mol Oncol. 2021 Feb 7;15(5):1486–506. doi: 10.1002/1878-0261.12903 (PMC8096796; doi:10.1002/1878-0261.12903)
Supplement: Supplementary file 12 — Table S2. List of angiogenesis‐related genes. [file MOL2-15-1486-s004.pdf]

Supplementary Table 2; List of angiogenesis related genes.

Gene name	Kit	ANGIOGENESIS	REGULATION_OF_ANGIOGENESIS	POSITIVE_REGULATION_OF_ANGIOGENESIS	NEGATIVE_REGULATION_OF_ANGIOGENESIS	HALLMARK_ANGIOGENESIS	GO_SPROUTING_ANGIOGENESIS	GO_POSITIVE_REGULATION_OF_SPROUTING_ANGIOGENESIS	GO_NEGATIVE_REGULATION_OF_SPROUTING_ANGIOGENESIS	GO_REGULATION_OF_CELL_MIGRATION_INVOLVED_IN_SPROUTING_ANGIOGENESIS	HELLEBREKERS_SILENCED_DURING_TUMOR_ANGIOGENESIS	ANGIOGENESIS (CST)
# of genes	55	48	26	10	13	36	189	66	80	85	77	81
ABI3BP											1	
ABL1							1	1		1		
ACSF2											1	
ACVRL1		1					1					
ADA											1	
ADAMTS1	1											1
ADAMTS9							1		1			
ADM											1	
ADTRP							1					
AGGF1		1	1	1								
AGTR1							1	1				
AKT1							1					
AKT3							1	1		1		
AMOT		1	1	1								
ANG	1	1										
ANGPT1	1						1					

ANGPT2	1											1
ANGPTL3		1	1	1								
ANGPTL4		1	1	1								
ANXA1							1	1		1		
APLNR							1	1				
APOE											1	
APOH						1						
APP						1						
AREG	1											
ARNT												1
ARTN	1											
ASAH1											1	
ASS1											1	
BDNF											1	
BMP4							1					
BMPER							1	1				
BTG1		1	1	1								
C1GALT1		1										
CA9												1
CADM1											1	
CANX		1										
CARD10							1		1	1		
CCBE1							1					
CCL11											1	
CCL2	1											1
CCL3	1											
CCND2						1						
CDH13		1					1					
CDH2											1	
CDH5												1
CDKN1A											1	
CEACAM1							1					
CHRNA7		1	1	1								
CIB1							1	1		1		
CLEC14A							1					
CLIP3											1	
CLU											1	
CNN1											1	
COL18A1	1											
COL2A1											1	
COL3A1						1						
COL4A2		1	1		1						1	
COL4A3		1	1		1							
COL5A2						1						
COL6A1											1	
CPE											1	
CREB3L1							1		1		1	

CREBBP												1
CSF2	1											
CTSB											1	
CUX1											1	
CXCL1											1	
CXCL16	1											
CXCL6						1					1	
CXCL8	1	1									1	
DKK3											1	
DLL1							1	1				
DLL4							1		1	1		1
DMD											1	
DPP4	1											
E2F2							1		1			
E2F7							1					
E2F8							1					
EDIL3											1	
EDN1	1											
EFNB1												1
EFNB2							1					1
EFNB3												1
EGF	1	1										
EGLN1												1
EGR3							1					
EMCN		1										
ENG	1											1
EP300												1
EPGN		1										
EPHA2							1					1
EPHB1												1
EPHB2												1
EPHB4							1					
EPN1							1		1			
EPN2							1		1			
ERAP1		1										
ESM1							1					
ETS1												1
F2RL1												1
F2RL2											1	
F3	1											1
FABP4											1	
FAT1											1	
FBN1											1	
FBXW7							1			1		
FGF1	1						1	1				1
FGF2	1						1	1		1		1
FGF4	1											

FGF7	1											
FGFBP1							1	1		1		
FGFR1						1						1
FGFR2												1
FGFR3												1
FGFR4												1
FILIP1											1	
FLT1							1					1
FLT4							1					1
FOXC2							1	1		1		
FOXO4		1	1		1							
FSTL1						1						
FTH1											1	
FUCA1											1	
GABRA1											1	
GADD45A											1	
GATA2							1	1		1		
GDNF	1											
GHRL							1	1				
GHSR							1	1				
GLUL							1					
GPLD1							1					
GREM1							1					1
HBEGF	1											
HDAC5							1		1	1		
HDAC7							1	1		1		
HDAC9							1	1		1		
HGF	1											1
HIF1A												1
HIF1AN												1
HLA-C											1	
HMOX1							1	1		1		1
HTATIP2		1	1									
ICAM1											1	
IER3											1	
IFI27											1	
IFI6											1	
IGF2R											1	
IGFBP1	1											
IGFBP2	1											
IGFBP3	1										1	
IGFBP7											1	
IL10							1	1				
IL17F		1	1		1							
IL18	1	1										
IL32											1	
IL6											1	

INHBA	1										1	
ITGA3											1	
ITGA5							1	1				1
ITGA6												1
ITGAV						1						1
ITGB1							1					1
ITGB1BP1							1		1	1		
ITGB3												1
ITGB5												1
ITM2B											1	
JAG1						1						1
JAG2						1						
JAK1							1	1				
JCAD							1	1		1		
JMJD6							1					
JMJD8							1	1				
KCNJ8						1						
KDR							1	1		1		1
KLF2							1		1			
KLF4							1	1	1	1		
LEF1							1					
LEP	1											
LOXL2							1					
LPL						1						
LRP1											1	
LRPAP1						1						
LUM						1						
MAP2K5							1		1	1		
MAP3K3							1	1		1		
MEFV											1	
MEOX2							1		1	1		
MIA3							1					
MIR101-1							1	1		1		
MIR101-2							1	1		1		
MIR10A							1	1		1		
MIR10B							1	1		1		
MIR1-1							1	1				
MIR1-2							1	1				
MIR125A							1	1				
MIR126							1	1		1		
MIR132							1	1		1		
MIR138-1							1		1			
MIR138-2							1		1			
MIR146A							1	1	1	1		
MIR150							1	1		1		
MIR155							1	1	1	1		
MIR15A							1		1	1		

MIR15B							1		1			
MIR16-1							1		1	1		
MIR16-2							1		1	1		
MIR17							1		1			
MIR188							1		1	1		
MIR18A							1		1			
MIR193A							1		1	1		
MIR196A1							1		1	1		
MIR196A2							1		1	1		
MIR19A							1		1			
MIR19B1							1		1	1		
MIR19B2							1		1	1		
MIR206							1		1	1		
MIR20A							1		1	1		
MIR22							1		1	1		
MIR221							1		1	1		
MIR222							1		1			
MIR2355							1		1	1		
MIR23A							1	1		1		
MIR23B							1	1	1	1		
MIR24-1							1		1			
MIR24-2							1		1			
MIR26A1							1		1	1		
MIR26A2							1		1	1		
MIR27A							1	1		1		
MIR27B							1	1		1		
MIR296							1	1		1		
MIR29C							1		1	1		
MIR30B							1	1				
MIR30C1							1		1			
MIR30C2							1		1			
MIR30E							1		1			
MIR31							1	1		1		
MIR320A							1		1	1		
MIR34A							1		1			
MIR34B							1		1			
MIR34C							1		1			
MIR375							1		1			
MIR377							1		1			
MIR410							1		1	1		
MIR494							1		1	1		
MIR495							1		1	1		
MIR503							1	1	1	1		
MIR7-2							1		1			
MIR7-3							1		1			
MIR92A1							1	1	1			
MIR92A2							1	1	1			

MIRLET7B							1	1				
MIRLET7F1							1	1		1		
MIRLET7F2							1	1		1		
MMP13												1
MMP2												1
MMP3												1
MMP7												1
MMP8	1											
MMP9	1											1
MMRN2							1		1	1		
MST1R												1
MSX1						1						
MX1											1	
MYH9		1										
NCL		1										
NDRG1												1
NDRG2												1
NDRG3												1
NDRG4											1	1
NEO1												1
NF1		1	1									
NGFR							1		1			
NNAT											1	
NOS3												1
NOTCH1							1		1	1		1
NOTCH2												1
NOTCH3												1
NOTCH4		1										1
NPC2											1	
NPPB		1	1		1						1	
NPR1		1	1		1							
NR2E1							1			1		
NR4A1							1					
NRARP							1					
NRG1	1											
NRP1						1	1	1		1		
NRP2												1
NUAK1											1	
OLFML3											1	
OLR1						1						
OTULIN							1					
P3H2											1	
PARVA							1					
PDCD10							1		1	1		
PDGFA	1					1						
PDGFB	1											
PDGFRA												1

PDGFRB												1
PDLIM3											1	
PDPK1							1	1				
PDPN												1
PECAM1												1
PF4	1	1	1		1	1						
PGF	1						1					
PGLYRP1						1						
PIK3C2A							1	1		1		
PIK3R3							1					
PKM							1	1				
PLAU	1											
PLG	1	1	1		1							
PLK2							1	1		1		
PLN												1
PML		1	1		1							
POSTN						1						
PPP1R16B							1	1				
PRG2						1						
PRL	1											
PROK1	1											
PROK2		1										
PROX1												1
PSPN	1											
PTGS2							1	1		1		1
PTK2						1						
PTK2B							1					
PTPN14												1
PTX3	1											
QSOX1											1	
RAMP2							1					
RECK							1					1
RELB											1	
REN												1
RHOA							1		1	1		
RHOB		1	1	1								
RHOJ							1	1		1		
RNH1		1	1									
ROBO1							1					
ROBO4		1										
RSPO3							1					
RUNX1		1	1	1								
S100A1							1	1				
S100A4						1						
SCG2		1										
SDC4											1	
SEMA3E							1					

SEMA5A							1					
SEMA6A							1		1			
SERPINA5						1						
SERPINB5	1											1
SERPINE1	1											
SERPINE2											1	
SERPINF1	1	1	1		1							
SHH		1										
SLCO1A2											1	
SLCO2A1						1						
SLIT2							1					
SMAD1							1	1				
SMTN											1	
SPHK1		1	1	1								
SPINK5		1	1		1							
SPP1						1						
SPRED1							1		1	1		
SPRY1												1
SRF							1					
SRPX2							1	1		1		
STAB1		1	1		1							
STARD13							1		1	1		
STC1						1						
SYNJ2BP							1		1			
TACSTD2											1	
TAGLN											1	
TBXA2R							1		1	1		
TDGF1							1					1
TEK							1					1
TFCP2												1
TGFA											1	1
TGFB1	1											1
TGFB2		1										1
TGFB3												1
TGFBR1												1
TGFBR3												1
THBD						1						
THBS1	1						1		1	1		1
THBS2	1											
THY1		1										
TIMP1	1					1						1
TIMP2												1
TIMP3												1
TIMP4	1											
TMEM45A											1	
TNC												1
TNFAIP3											1	

TNFRSF21						1						
TNFSF12		1	1	1								
TNNI3		1	1		1							
TSPAN2											1	
TYMP	1											1
UCP2											1	
UNC5B												1
USF1											1	
VASH1	1											
VAV2						1						
VCAN						1					1	
VEGF	1											
VEGFA		1				1	1	1		1		
VEGFB							1					1
VEGFC	1						1					1
VEGFD							1					
VTN						1						
VWF												1
