# Supplementary material for: REST promotes ETS1‐dependent vascular growth in medulloblastoma
Source: Mol Oncol. 2021 Feb 7;15(5):1486–506. doi: 10.1002/1878-0261.12903 (PMC8096796; doi:10.1002/1878-0261.12903)
Supplement: Supplementary file 13 — Table S3. List of differentially expressed genes between high‐REST and low‐REST patient tumors (volcano plot). [file MOL2-15-1486-s007.pdf]

Supplementary Table 3; List of differentially expressed genes between high-REST patients and low-REST patients (volcano plot).

Gene name	Protein array	Exp diff (HR-LR)	p-value	-log10(p-value)
PTPN14		0.86	9.43E-14	13.03
ACSF2		0.75	2.19E-13	12.66
IGF2R		0.80	3.36E-10	9.47
SPRED1		0.61	5.89E-09	8.23
NOTCH3		0.80	8.72E-09	8.06
DLL1		0.53	2.63E-08	7.58
NRP2		0.57	3.18E-08	7.50
NOTCH2		0.35	5.50E-08	7.26
E2F8		0.72	5.80E-08	7.24
GABRA1		-0.70	1.11E-07	6.95
TNFAIP3		0.64	1.80E-07	6.75
JAG1		0.66	1.88E-07	6.73
E2F7		0.64	1.91E-07	6.72
LOXL2		0.65	2.11E-07	6.68
GADD45A		0.46	3.06E-07	6.51
MIR24-1		-0.65	7.55E-07	6.12
ANGPT2	1	0.68	7.97E-07	6.10
COL4A2		0.62	1.07E-06	5.97
EPHB4		0.36	1.49E-06	5.83
PGF	1	0.86	1.51E-06	5.82
COL5A2		0.60	1.72E-06	5.76
SCG2		-0.63	1.77E-06	5.75
PARVA		0.65	1.78E-06	5.75
TGFB3		0.51	1.87E-06	5.73
FSTL1		0.43	2.23E-06	5.65
ETS1		0.65	2.55E-06	5.59
ITGA5		0.62	3.86E-06	5.41
NGFR		-0.51	4.24E-06	5.37
ADA		0.62	5.17E-06	5.29
PTK2B		-0.75	9.91E-06	5.00
CPE		-0.44	1.49E-05	4.83
HDAC7		0.49	1.59E-05	4.80
PECAM1		0.59	1.68E-05	4.77
MIR27B		-0.55	2.24E-05	4.65
EGF	1	0.40	4.57E-05	4.34
NDRG4		-0.44	4.69E-05	4.33
PML		0.59	5.08E-05	4.29
HIF1A		0.51	5.48E-05	4.26
MIR30C2		0.52	7.39E-05	4.13
CLIP3		-0.26	8.26E-05	4.08
NOTCH1		0.56	9.30E-05	4.03
DPP4	1	-0.67	1.08E-04	3.97
STC1		0.35	1.09E-04	3.96
MIR23B		-0.50	1.66E-04	3.78
FLT1		0.52	1.74E-04	3.76

IGFBP7		0.53	1.87E-04	3.73
EFNB2		0.49	1.94E-04	3.71
SDC4		0.38	2.78E-04	3.56
VWF		0.52	2.99E-04	3.52
KDR		0.44	3.03E-04	3.52
NOTCH4		0.53	3.08E-04	3.51
CDH5		0.48	3.18E-04	3.50
ANG	1	0.47	3.55E-04	3.45
SMAD1		-0.27	4.53E-04	3.34
ESM1		0.33	4.99E-04	3.30
MIA3		0.38	5.13E-04	3.29
DLL4		0.52	6.14E-04	3.21
EPHA2		0.21	6.47E-04	3.19
COL18A1	1	0.50	6.57E-04	3.18
VEGFC	1	0.34	7.12E-04	3.15
IL32		0.46	7.25E-04	3.14
UCP2		0.45	7.27E-04	3.14
SLCO1A2		-0.48	7.42E-04	3.13
FILIP1		0.43	7.57E-04	3.12
VCAN		0.38	9.09E-04	3.04
SLCO2A1		0.47	9.16E-04	3.04
ROBO4		0.35	1.00E-03	3.00
EFNB3		-0.36	1.01E-03	2.99
AREG	1	0.38	1.11E-03	2.96
PGLYRP1		-0.40	1.22E-03	2.91
FGFR4		-0.40	1.37E-03	2.86
SRF		0.37	1.39E-03	2.86
MYH9		0.40	1.47E-03	2.83
RHOB		-0.35	1.58E-03	2.80
PROX1		0.24	1.59E-03	2.80
ADAMTS1	1	0.36	1.59E-03	2.80
IGFBP3	1	0.45	1.73E-03	2.76
FGF2	1	0.28	1.92E-03	2.72
MIR155		0.40	1.97E-03	2.71
HDAC9		-0.20	2.04E-03	2.69
STARD13		0.32	2.08E-03	2.68
CCBE1		0.16	2.26E-03	2.65
ACVRL1		0.40	2.43E-03	2.61
MIR15B		0.40	2.70E-03	2.57
TGFB1	1	0.27	2.87E-03	2.54
ITGB1		0.35	2.94E-03	2.53
FABP4		0.41	3.05E-03	2.52
BTG1		-0.34	3.17E-03	2.50
GDNF	1	-0.21	3.51E-03	2.45
LUM		0.29	3.55E-03	2.45
ADAMTS9		0.30	3.78E-03	2.42
PRG2		0.38	3.80E-03	2.42
LEF1		-0.29	3.86E-03	2.41
SMTN		0.33	4.07E-03	2.39

CCND2			0.48	4.67E-03	2.33
MMRN2			0.38	4.91E-03	2.31
TGFA			-0.19	5.32E-03	2.27
PLK2			0.28	5.72E-03	2.24
SERPINE2			-0.37	5.81E-03	2.24
CLEC14A			0.38	6.16E-03	2.21
MMP7			0.28	6.34E-03	2.20
HMOX1			0.33	6.83E-03	2.17
MIR495			-0.29	7.26E-03	2.14
MIR15A			0.36	7.61E-03	2.12
MIR221			0.35	7.83E-03	2.11
EPGN			0.37	8.62E-03	2.06
INHBA	1		0.34	8.89E-03	2.05
ETV4			0.29	9.00E-03	2.05
SLIT2			0.28	9.32E-03	2.03
ABL1			0.27	9.51E-03	2.02
PTX3	1		0.28	1.01E-02	2.00
TNFRSF21			0.26	1.02E-02	1.99
F2RL1			0.38	1.02E-02	1.99
IL18	1		0.36	1.03E-02	1.99
IL6			0.32	1.04E-02	1.98
TEK			0.33	1.11E-02	1.96
CSF2	1		-0.36	1.18E-02	1.93
THBS1	1		0.27	1.19E-02	1.92
JAG2			0.25	1.24E-02	1.91
TIMP2			0.33	1.25E-02	1.90
MMP9	1		0.37	1.27E-02	1.89
CHRNA7			0.29	1.31E-02	1.88
UNC5B			-0.28	1.34E-02	1.87
BDNF			-0.40	1.36E-02	1.87
ITGB5			0.29	1.41E-02	1.85
GPLD1			-0.24	1.42E-02	1.85
MIR410			-0.29	1.45E-02	1.84
MIR34A			0.37	1.46E-02	1.84
PDGFA	1		0.16	1.57E-02	1.80
MIR29C			-0.32	1.68E-02	1.77
MIR126			0.32	1.71E-02	1.77
SERPINA5			0.32	1.86E-02	1.73
AMOT			0.24	1.89E-02	1.72
MIR138-2			-0.18	1.90E-02	1.72
PDPN			0.26	2.00E-02	1.70
COL2A1			0.10	2.03E-02	1.69
FGFR2			0.30	2.05E-02	1.69
SPRY1			0.27	2.35E-02	1.63
QSOX1			0.32	2.38E-02	1.62
PKM			0.19	2.40E-02	1.62
OTULIN			0.28	2.41E-02	1.62
NDRG3			-0.33	2.43E-02	1.61
ABI3BP			0.29	2.44E-02	1.61

CARD10		0.15	2.45E-02	1.61
ANGPTL4		0.35	2.56E-02	1.59
MIR377		-0.24	2.58E-02	1.59
LEP	1	-0.28	2.61E-02	1.58
F2RL2		-0.37	2.66E-02	1.57
BMPER		-0.34	2.68E-02	1.57
MIR17		0.30	2.72E-02	1.57
MIR18A		0.30	2.72E-02	1.57
MIR19A		0.30	2.72E-02	1.57
MIR19B1		0.30	2.72E-02	1.57
MIR20A		0.30	2.72E-02	1.57
MIR92A1		0.30	2.72E-02	1.57
DKK3		-0.36	2.73E-02	1.56
VEGFA		0.29	2.76E-02	1.56
VEGFA	1	0.33	2.78E-02	1.56
TBXA2R		0.32	2.83E-02	1.55
PDCD10		0.31	2.85E-02	1.54
MST1R		-0.28	2.86E-02	1.54
RHOA		0.20	2.90E-02	1.54
ENG	1	0.28	3.11E-02	1.51
MMP13		0.31	3.11E-02	1.51
TIMP1	1	0.25	3.13E-02	1.50
POSTN		0.23	3.32E-02	1.48
DMD		-0.16	3.38E-02	1.47
EMCN		0.29	3.39E-02	1.47
COL3A1		0.22	3.48E-02	1.46
MIR26A1		-0.28	3.49E-02	1.46
EGLN1		0.27	3.62E-02	1.44
EPN2		-0.25	3.72E-02	1.43
NUAK1		0.25	3.93E-02	1.41
NNAT		0.23	3.97E-02	1.40
TIMP3		0.24	4.02E-02	1.40
E2F2		0.26	4.03E-02	1.40
THBS2	1	0.25	4.31E-02	1.37
RNH1		-0.26	4.58E-02	1.34
RHOJ		-0.26	4.69E-02	1.33
MIR150		-0.26	4.79E-02	1.32
FBXW7		-0.39	4.82E-02	1.32
MIR22		-0.26	4.86E-02	1.31
